# Supplementary material for: BayesCCE: a Bayesian framework for estimating cell-type composition from DNA methylation without the need for methylation reference
Source: Genome Biol. 2018 Sep 21;19:141. doi: 10.1186/s13059-018-1513-2 (PMC6151042; doi:10.1186/s13059-018-1513-2)
Supplement: Supplementary file 1 — Supplementary Tables and Figures. (PDF 1277 kb) [file 13059_2018_1513_MOESM1_ESM.pdf]

Additional file 1: Supplementary Tables and Figures

BayesCCE: a Bayesian framework for estimating cell-type composition from DNA methylation without the need for methylation reference

Elior Rahmani, Regev Schweiger, Liat Shenhav, Theodora Wingert, Ira Hofer, Eilon Gabel, Eleazar Eskin and Eran Halperin.

		Absolute Correlation								
		$k = 3$			$k = 6$					
data set	Method	Gran	Lymph	Mono	Gran	CD4+	CD8+	B	NK	Mono
Hannum et al. [1]	ReFACTor	0.166	0.975	0.051	0.95	0.389	0.335	0.34	0.129	0.201
	NNMF	0.952	0.938	0.172	0.85	0.3	0.184	0.644	0.077	0.118
	MeDeCom	0.448	0.923	0.285	0.631	0.505	0.351	0.433	0.015	0.258
	BayesCCE	0.936	0.872	0.251	0.921	0.703	0.575	0.559	0.326	0.405
	BayesCCE impute	0.965	0.988	0.516	0.951	0.851	0.626	0.899	0.636	0.403
	BayesCCE impute ext	0.959	0.985	0.214	0.957	0.804	0.513	0.744	0.474	0.103
Liu et al. [2]	ReFACTor	0.164	0.982	0.105	0.961	0.089	0.495	0.338	0.137	0.309
	NNMF	0.936	0.98	0.092	0.902	0.269	0.588	0.023	0.089	0.328
	MeDeCom	0.97	0.773	0.054	0.73	0.563	0.24	0.16	0.283	0.293
	BayesCCE	0.971	0.956	0.021	0.973	0.785	0.719	0.59	0.487	0.209
	BayesCCE impute	0.977	0.986	0.561	0.982	0.792	0.675	0.609	0.554	0.496
	BayesCCE impute ext	0.988	0.986	0.529	0.971	0.726	0.66	0.646	0.516	0.483
Hannon et al. I [3]	ReFACTor	0.387	0.919	0.025	0.883	0.013	0.403	0.358	0.043	0.147
	NNMF	0.916	0.959	0.157	0.682	0.597	0.401	0.159	0.074	0.193
	MeDeCom	0.934	0.7	0.027	0.801	0.342	0.285	0.297	0.16	0.135
	BayesCCE	0.947	0.973	0.266	0.956	0.628	0.297	0.451	0.186	0.153
	BayesCCE impute	0.938	0.977	0.305	0.944	0.738	0.467	0.643	0.366	0.35
	BayesCCE impute ext	0.971	0.973	0.528	0.967	0.665	0.355	0.687	0.384	0.419
Hannon et al. II [3]	ReFACTor	0.106	0.977	0.072	0.952	0.011	0.214	0.427	0.429	0.05
	NNMF	0.833	0.805	0.14	0.598	0.416	0.245	0.234	0.038	0.143
	MeDeCom	0.829	0.724	0.018	0.482	0.329	0.222	0.107	0.124	0.102
	BayesCCE	0.91	0.981	0.217	0.914	0.713	0.316	0.425	0.206	0.107
	BayesCCE impute	0.973	0.983	0.441	0.965	0.756	0.62	0.823	0.641	0.519
	BayesCCE impute ext	0.957	0.98	0.299	0.972	0.751	0.563	0.775	0.618	0.604

Table S1: A summary of the correlation of existing reference-free methods and BayesCCE with each cell type in four whole-blood data sets (considering reference-based estimates as the ground truth), under the assumption of six constituting cell types in blood ($k = 6$): granulocytes, monocytes and four subtypes of lymphocytes (CD4+, CD8+, B cells and NK cells), and under the assumption of three constituting cell types in blood ($k = 3$): granulocytes, monocytes and lymphocytes. For each of the methods, ReFACTor, NNMF, MeDeCom and BayesCCE, we considered a single component per cell type (see Methods). In addition, we considered the scenario wherein cell counts are known for 5% of the samples (BayesCCE imp), and the scenario wherein samples from external data with both methylation levels and cell counts are available (5% of the smaple size; BayesCCE imp ext). For BayesCCE imp and BayesCCE imp ext, correlations were calculated after excluding the samples with assumed known cell counts.

		Mean Absolute Error								
		$k = 3$			$k = 6$					
data set	Method	Gran	Lymph	Mono	Gran	CD4+	CD8+	B	NK	Mono
Hannum et al. [1]	ReFACTor	0.187	0.104	0.587	0.233	0.498	0.335	0.627	0.593	0.161
	NNMF	0.113	0.11	0.067	0.141	0.121	0.062	0.272	0.051	0.046
	MeDeCom	0.232	0.064	0.276	0.445	0.072	0.125	0.148	0.134	0.106
	BayesCCE	0.237	0.186	0.114	0.501	0.097	0.166	0.041	0.043	0.422
	BayesCCE impute	0.022	0.022	0.021	0.022	0.027	0.029	0.015	0.023	0.021
	BayesCCE impute ext	0.044	0.018	0.046	0.032	0.042	0.032	0.031	0.037	0.027
Liu et al. [2]	ReFACTor	0.183	0.14	0.54	0.233	0.356	0.497	0.41	0.423	0.317
	NNMF	0.197	0.196	0.046	0.223	0.082	0.276	0.042	0.049	0.058
	MeDeCom	0.284	0.193	0.202	0.398	0.071	0.079	0.1	0.165	0.108
	BayesCCE	0.23	0.214	0.043	0.094	0.034	0.038	0.049	0.076	0.038
	BayesCCE impute	0.023	0.015	0.017	0.02	0.033	0.034	0.016	0.027	0.018
	BayesCCE impute ext	0.013	0.016	0.021	0.019	0.032	0.045	0.021	0.03	0.019
Hannon et al. I [3]	ReFACTor	0.222	0.131	0.383	0.201	0.318	0.284	0.445	0.404	0.437
	NNMF	0.218	0.221	0.045	0.463	0.221	0.305	0.05	0.046	0.043
	MeDeCom	0.215	0.151	0.246	0.408	0.062	0.083	0.117	0.122	0.115
	BayesCCE	0.27	0.23	0.084	0.311	0.159	0.053	0.054	0.066	0.042
	BayesCCE impute	0.022	0.014	0.023	0.034	0.027	0.028	0.014	0.026	0.016
	BayesCCE impute ext	0.014	0.03	0.017	0.017	0.03	0.03	0.027	0.026	0.016
Hannon et al. II [3]	ReFACTor	0.231	0.199	0.368	0.185	0.363	0.272	0.39	0.223	0.28
	NNMF	0.468	0.47	0.048	0.502	0.086	0.624	0.039	0.048	0.061
	MeDeCom	0.207	0.08	0.277	0.413	0.082	0.097	0.131	0.123	0.125
	BayesCCE	0.205	0.191	0.064	0.31	0.192	0.034	0.07	0.07	0.038
	BayesCCE impute	0.013	0.012	0.015	0.027	0.025	0.025	0.011	0.023	0.015
	BayesCCE impute ext	0.017	0.015	0.035	0.014	0.026	0.027	0.015	0.022	0.016

Table S2: A summary of the mean absolute error of existing reference-free methods and BayesCCE with each cell type in four whole-blood data sets (considering reference-based estimates as the ground truth), under the assumption of six constituting cell types in blood ($k = 6$): granulocytes, monocytes and four subtypes of lymphocytes (CD4+, CD8+, B cells and NK cells), and under the assumption of three constituting cell types in blood ($k = 3$): granulocytes, monocytes and lymphocytes. For each of the methods, ReFACTor, NNMF, MeDeCom and BayesCCE, we considered a single component per cell type (see Methods). In addition, we considered the scenario wherein cell counts are known for 5% of the samples (BayesCCE imp), and the scenario wherein samples from external data with both methylation levels and cell counts are available (5% of the smaple size; BayesCCE imp ext). For BayesCCE imp and BayesCCE imp ext, absolute errors were calculated after excluding the samples with assumed known cell counts.

	data set	Method	Single prior		Stratified prior	
			MAC	MAE	MAC	MAE
$k = 3$	Hannum et al. [1] (Aging)	BayesCCE	0.661	0.102	0.667	0.105
		BayesCCE impute	0.829	0.022	0.830	0.021
	Liu et al. [2] (Rheumatoid arthritis)	BayesCCE	0.685	0.094	0.681	0.040
		BayesCCE impute	0.893	0.014	0.894	0.014
	Hannon et al. I [3] (Schizophrenia)	BayesCCE	0.632	0.111	0.633	0.111
		BayesCCE impute	0.784	0.017	0.785	0.016
	Hannon et al. II [3] (Schizophrenia)	BayesCCE	0.490	0.252	0.492	0.206
		BayesCCE impute	0.815	0.012	0.816	0.012
$k = 6$	Hannum et al. [1] (Aging)	BayesCCE	0.497	0.113	0.510	0.114
		BayesCCE impute	0.718	0.026	0.654	0.027
	Liu et al. [2] (Rheumatoid arthritis)	BayesCCE	0.537	0.041	0.557	0.058
		BayesCCE impute	0.711	0.024	0.697	0.023
	Hannon et al. I [3] (Schizophrenia)	BayesCCE	0.463	0.172	0.436	0.164
		BayesCCE impute	0.601	0.022	0.602	0.022
	Hannon et al. II [3] (Schizophrenia)	BayesCCE	0.485	0.086	0.471	0.075
		BayesCCE impute	0.603	0.023	0.613	0.024

Table S3: A summary of the performance of BayesCCE using a single prior versus using a separate prior for cases and controls (stratified prior). Mean absolute correlation (MAC) and mean absolute error (MAE) values are presented under the assumption of six constituting cell types in blood ($k = 6$): granulocytes, monocytes and four subtypes of lymphocytes (CD4+, CD8+, B cells and NK cells), and under the assumption of three constituting cell types in blood ($k = 3$): granulocytes, monocytes and lymphocytes. A standard application of BayesCCE was compared with the scenario wherein cell counts are known for 5% of the samples (BayesCCE imp). In the later case, correlations were calculated after excluding the samples with assumed known cell counts. For the Hannum et al. data set, cases were defined as individuals with age above the median age in the study. For each data set, each of the calculated priors (the single general prior, the cases only prior and the controls only prior) was estimated using 5% of the samples in the data, which were then excluded from the subsequent analysis.

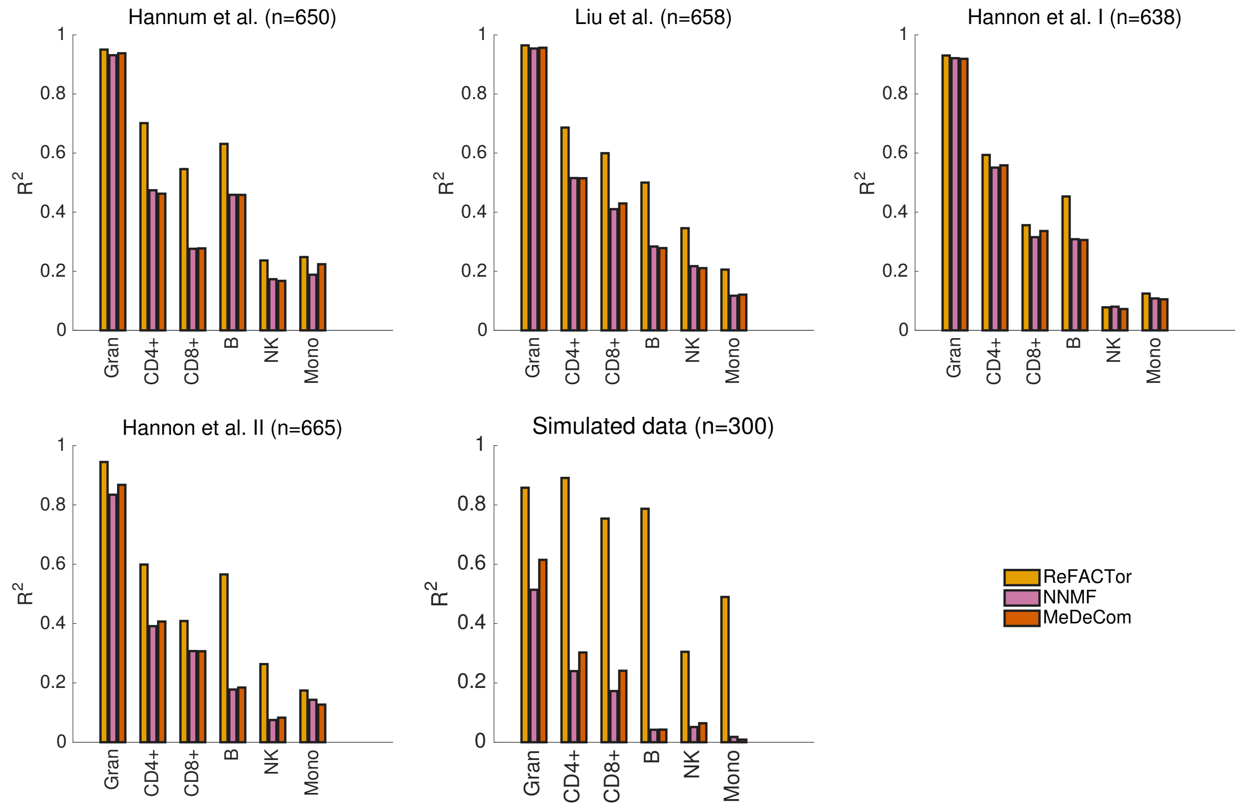

Figure S1: The fraction of cell type composition variance explained (R^2) by several reference-free methods. For each of the different methods, ReFACTor, NNMF and MeDeCom, a linear model was fitted for each of the six cell types using six components. The results presented for the simulated data were averaged across ten different simulated data sets.

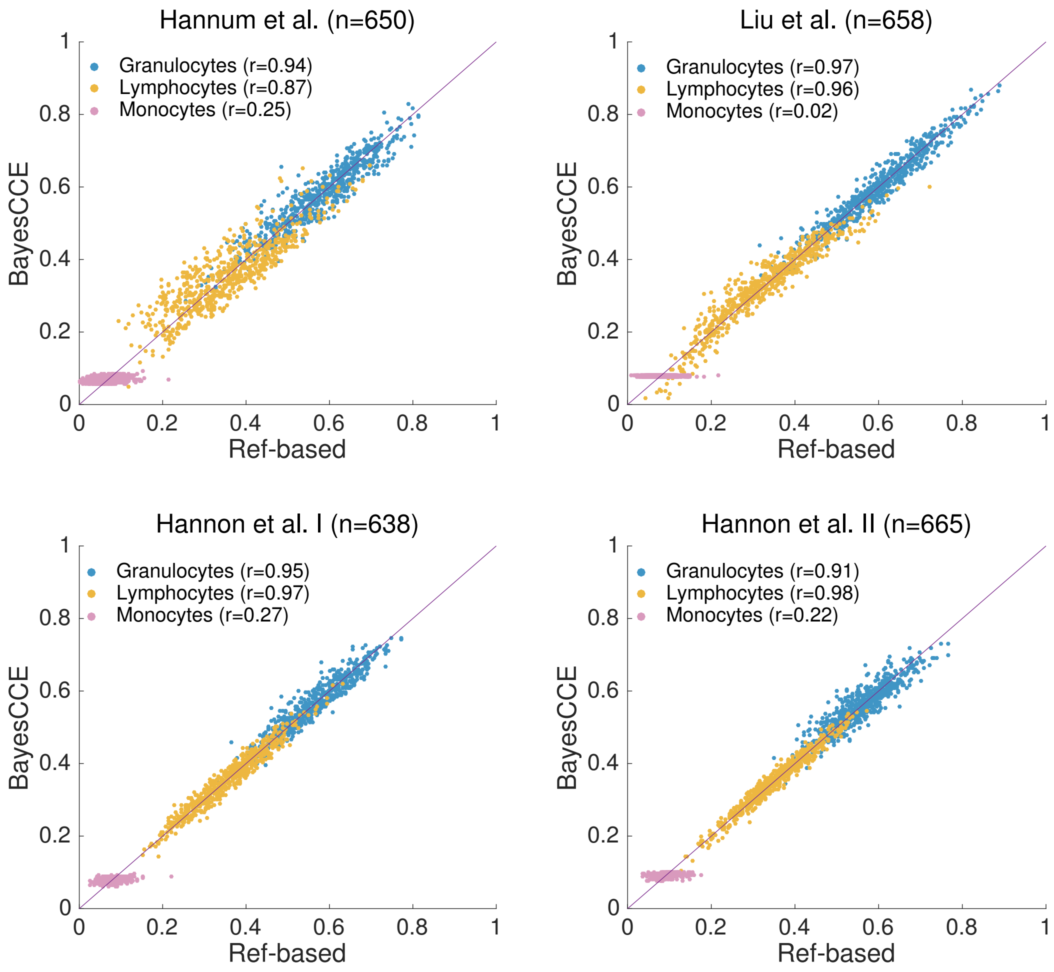

Figure S2: BayesCCE captures cell type proportions in four data sets under the assumption of three constituting cell types in blood ($k = 3$): granulocytes, monocytes and lymphocytes. The BayesCCE estimated components were linearly transformed to match their corresponding cell types in scale (see Methods).

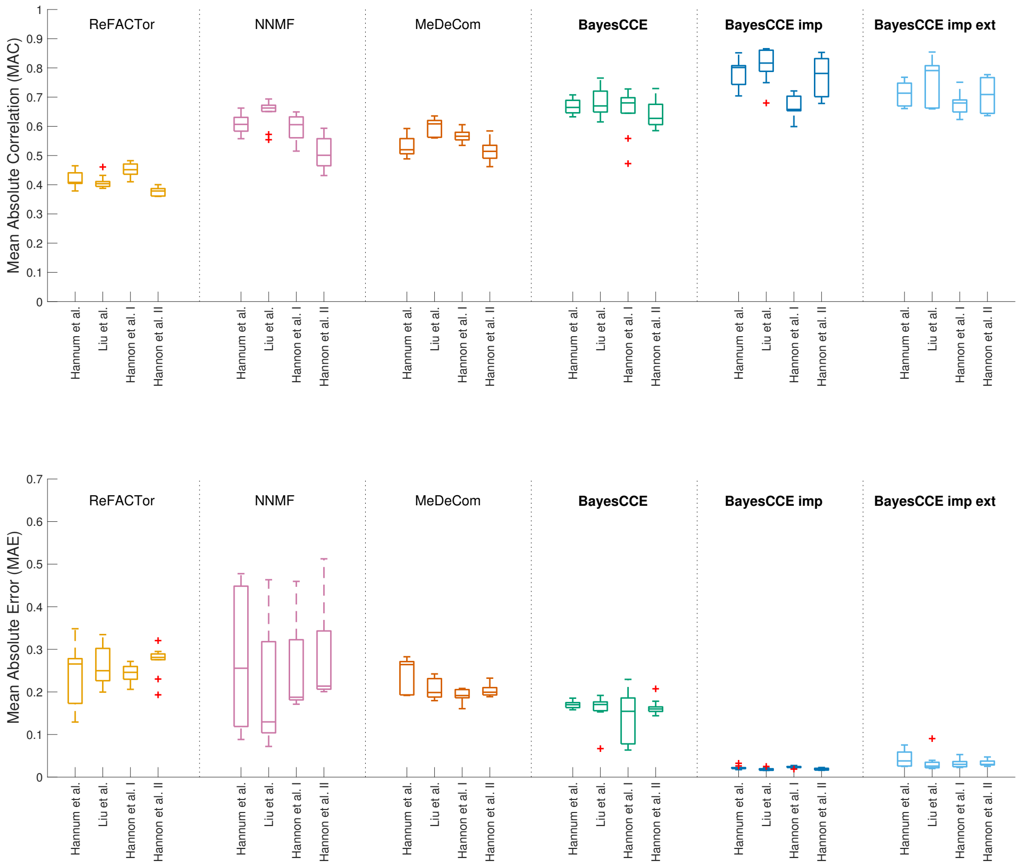

Figure S3: The performance of existing reference-free methods and BayesCCE under the assumption of three constituting cell types in blood ($k = 3$): granulocytes, monocytes and lymphocytes. For each method, box plots show for each data set the performance across ten sub-sampled data sets ($n = 300$), with the median indicated by a horizontal line. For each of the methods, ReFACTor, NNMF, MeDeCom and BayesCCE, we considered a single component per cell type (see Methods). Additionally, we considered the scenario of cell counts imputation wherein cell counts were known for 5% of the samples ($n = 15$; BayesCCE imp), and the scenario wherein samples from external data with both methylation levels and cell counts were used in the analysis ($n = 15$; BayesCCE imp ext). Top panel: mean absolute correlation (MAC) across all cell types. Bottom panel: mean absolute error (MAE) across all cell types. For BayesCCE imp and BayesCCE imp ext, the MAC and MAE values were calculated while excluding the samples with assumed known cell counts.

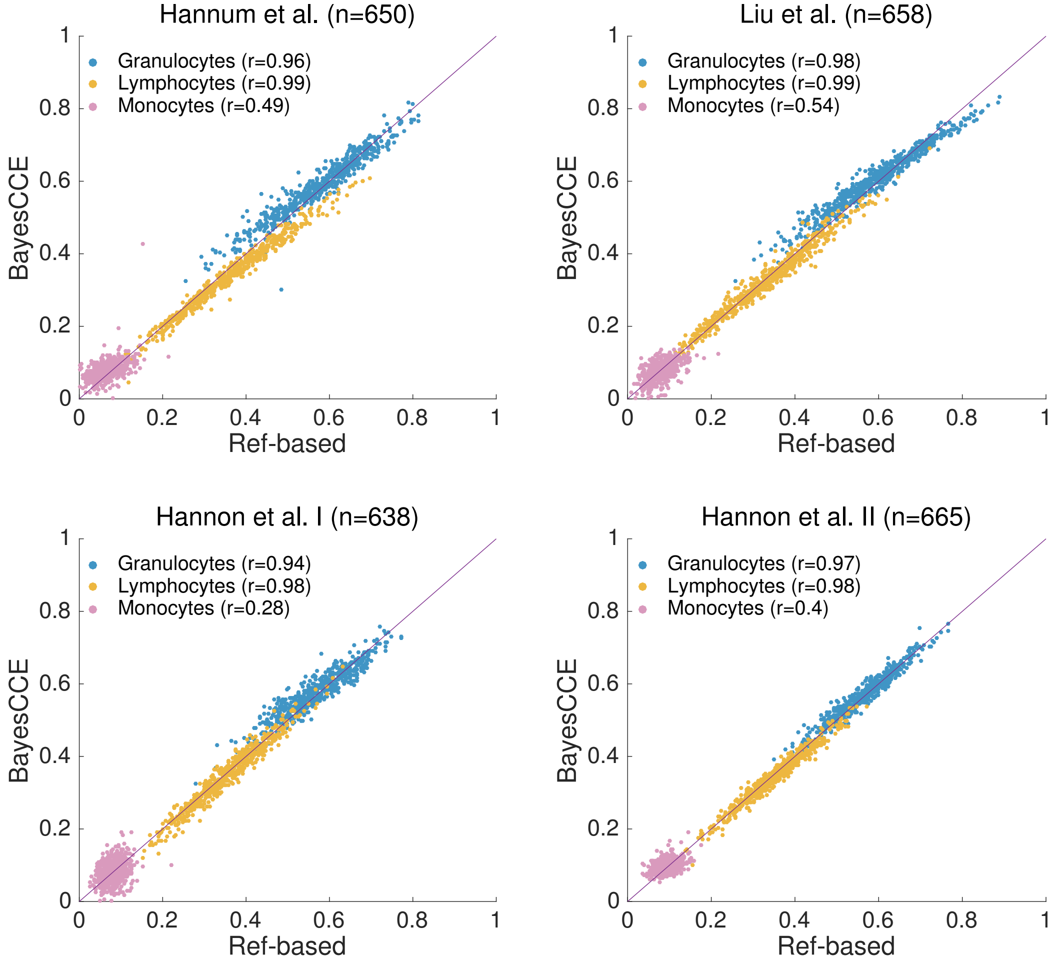

Figure S4: BayesCCE captures cell type proportions in four data sets under the assumption of three constituting cell types in blood ($k = 3$): granulocytes, monocytes and lymphocytes, and assuming known cell counts for randomly selected 5% of the samples in the data. All correlations were calculated while excluding the samples with assumed known cell counts.

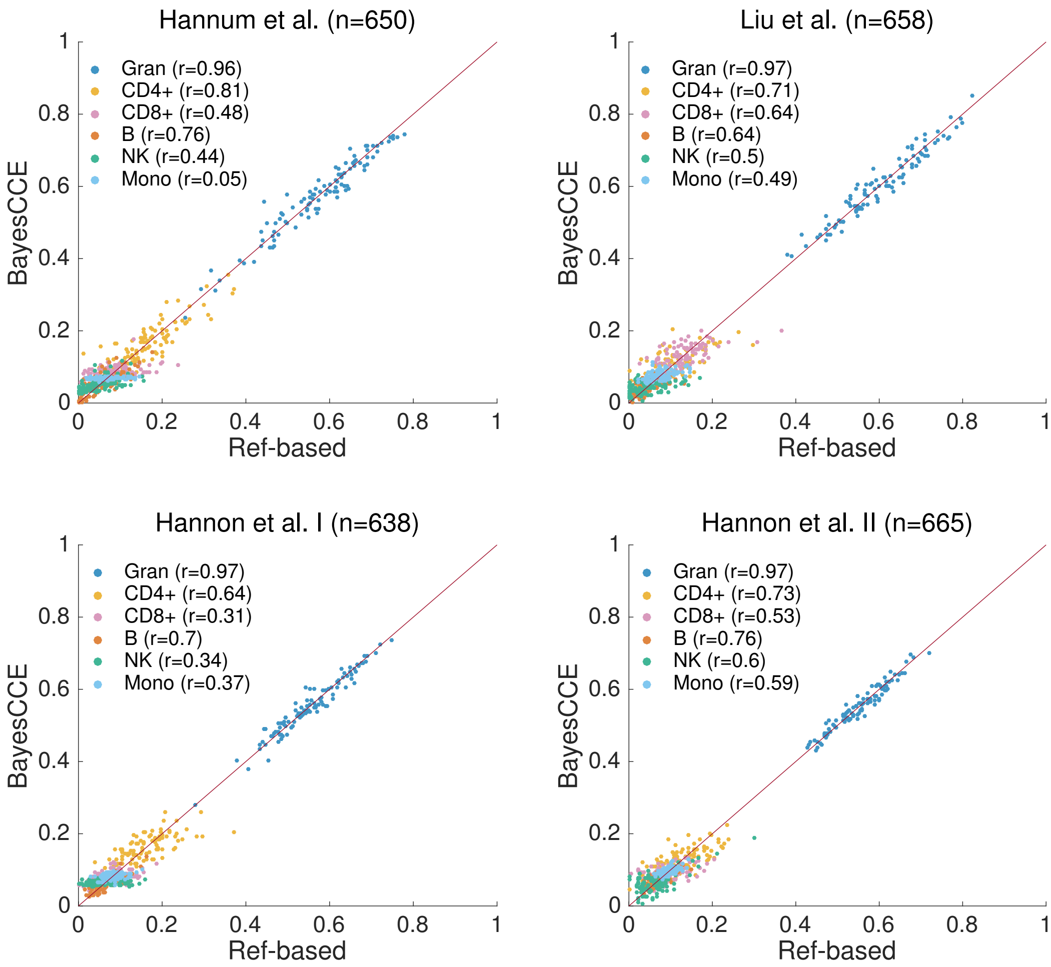

Figure S5: BayesCCE captures cell type proportions in four data sets under the assumption of six constituting cell types in blood ($k = 6$): granulocytes, monocytes and four subtypes of lymphocytes (CD4+, CD8+, B cells and NK cells), and including a group of samples with known cell counts from external data. For each data set, samples from one of the other data sets were included in the analysis (5% of the sample size), while assuming that both their methylation levels and cell counts are known. All correlations were calculated while excluding the samples with assumed known cell counts. For convenience of visualization, we only plot the results of 100 randomly selected samples for each data set.

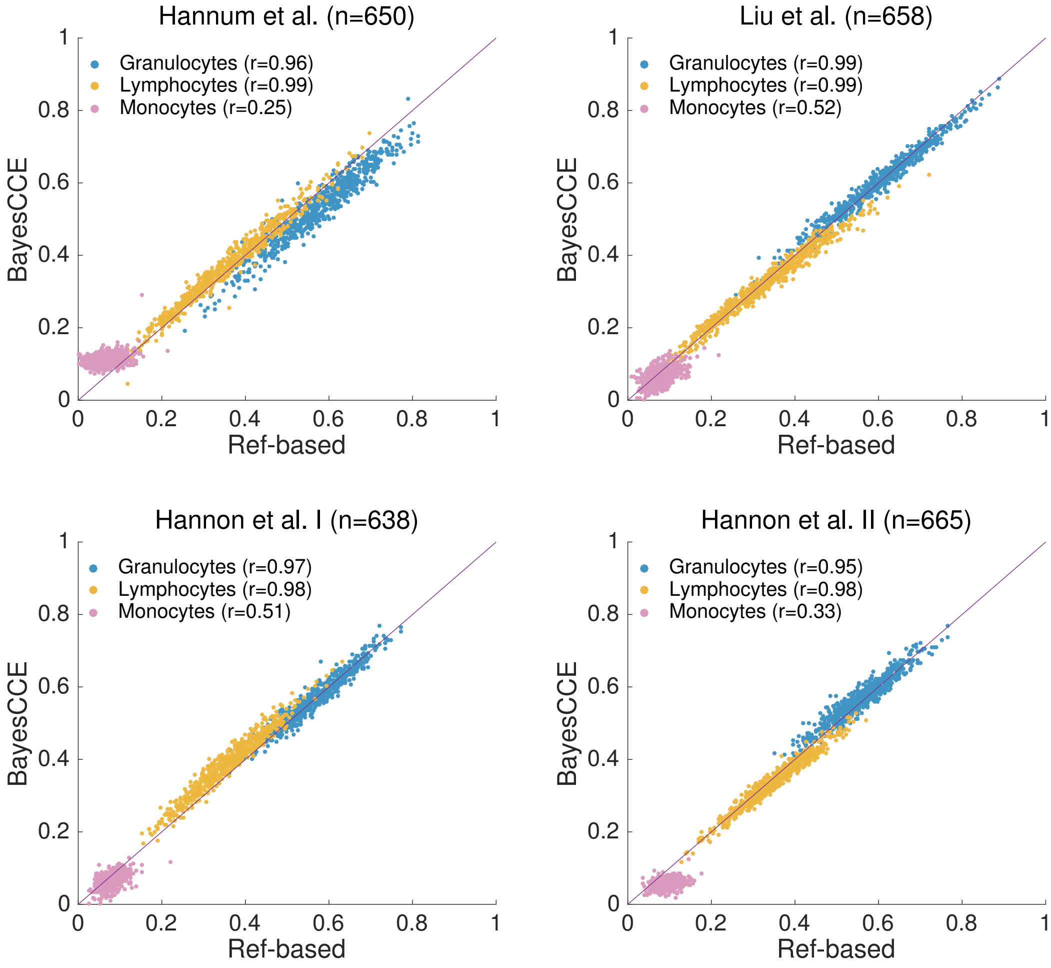

Figure S6: BayesCCE captures cell type proportions in four data sets under the assumption of three constituting cell types in blood ($k = 3$): granulocytes, monocytes and lymphocytes, and including a group of samples with known cell counts from external data. For each data set, samples from one of the other data sets were included in the analysis (5% of the sample size), while assuming that both their methylation levels and cell counts are known. All correlations were calculated while excluding the samples with assumed known cell counts.

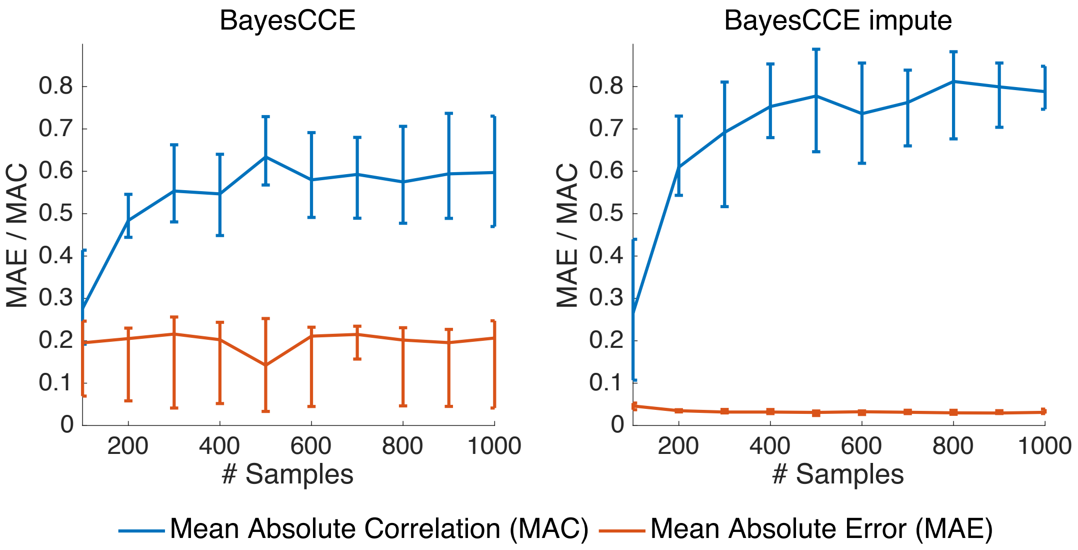

Figure S7: Performance of BayesCCE without known cell counts and BayesCCE with known cell counts (BayesCCE imp) for 15 of the samples as a function of the number of samples in simulated data ($k = 6$). Presented are the medians of the mean absolute correlation values (MAC; in blue) and the medians of the mean absolute error values (MAE; in red) across the six cell types. Error bars indicate the range of MAC and MAE values across ten different executions for each sample size. In BayesCCE imp, all MAC and MAE values were calculated while excluding the samples with assumed known cell counts.

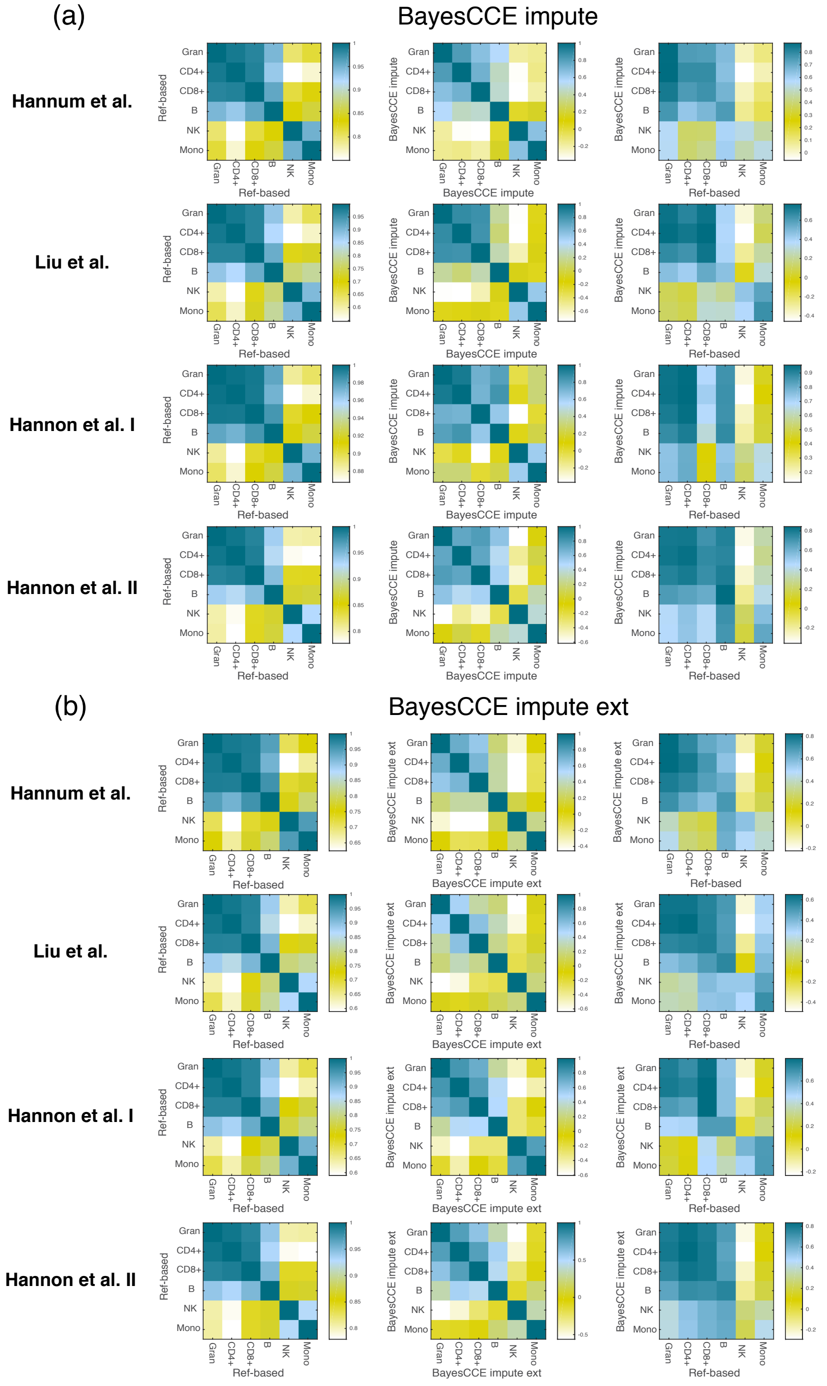

Figure S8: Correlation maps of the estimated cell-type-specific methylomes using BayesCCE under the assumption of six constituting cell types in blood ($k = 6$): granulocytes, monocytes and four subtypes of lymphocytes (CD4+, CD8+, B cells and NK cells). (a) For each of four data sets, correlation maps were calculated using cell-type-specific mean methylation levels estimated from a reference data set of methylation levels collected from sorted blood cell types by Reinius et al. (left column), using the estimates obtained by BayesCCE under the assumption of known cell counts for 5% of the samples (BayesCCE imp; middle column), and using the reference-based estimates versus the BayesCCE estimates (right column). (b) Similar to (a), only this time using BayesCCE in a scenario wherein samples from external data with both methylation levels and cell counts were available (5% of the sample size; BayesCCE imp ext).

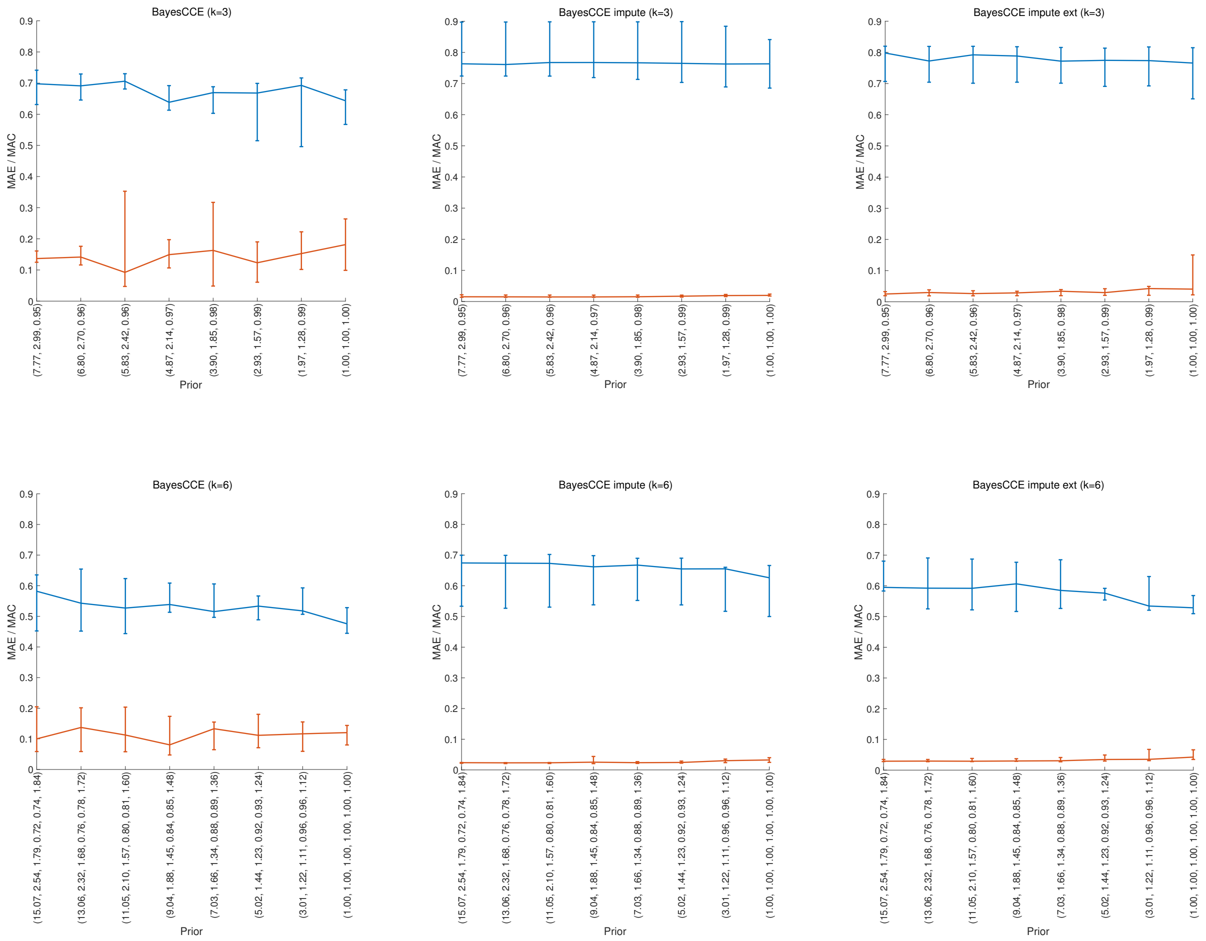

Figure S9: The performance of BayesCCE as a function of increasing noise introduced by the prior information, under the assumption of three constituting cell types in blood ($k = 3$): granulocytes, monocytes and lymphocytes (top panel), and under the assumption of six constituting cell types in blood ($k = 6$): granulocytes, monocytes and four subtypes of lymphocytes (CD4+, CD8+, B cells and NK cells; bottom panel). In this experiment, we evaluated BayesCCE, BayesCCE in a scenario wherein cell counts are known for 5% of the samples in the data (BayesCCE imp), and BayesCCE in a scnario wherein cell counts and methylation levels for samples from external data are included in the analysis (5% of the sample size; BayesCCE imp ext). For each method, presented are the values of mean absolute correlation (MAC) and mean absolute error (MAE) across all cell types as a function of the noise introduced into the prior information. Error bars indicate the performance across four data sets: Hannum et al. [1], Liu et al. [2], Hannon et al. I, and Hannon et al. II [3]. The range of the prior information was set between the prior estimated from real blood cell counts (see Methods) and a non-informative prior (a vector of ones).

References

[1] Hannum, G. *et al.* Genome-wide methylation profiles reveal quantitative views of human aging rates. *Molecular cell* **49**, 359–367 (2013).

[2] Liu, Y. *et al.* Epigenome-wide association data implicate dna methylation as an intermediary of genetic risk in rheumatoid arthritis. *Nature biotechnology* **31**, 142–147 (2013).

[3] Hannon, E. *et al.* An integrated genetic-epigenetic analysis of schizophrenia: evidence for co-localization of genetic associations and differential dna methylation. *Genome biology* **17**, 176 (2016).
