# Supplementary material for: BayesCCE: a Bayesian framework for estimating cell-type composition from DNA methylation without the need for methylation reference
Source: Genome Biol. 2018 Sep 21;19:141. doi: 10.1186/s13059-018-1513-2 (PMC6151042; doi:10.1186/s13059-018-1513-2)
Supplement: Supplementary file 2 — Review history. (DOCX 91 kb) [file 13059_2018_1513_MOESM2_ESM.docx]

Review History

**First round of review**

**Reviewer 1**

**Are the methods appropriate to the aims of the study, are they well described, and are necessary controls included?**
The methods are appropriate and well described.

**Are the conclusions adequately supported by the data shown?**

No. While the presented results are convincing and have a solid mathematical foundation, there are several issues with the general setup and the way certain analysis were performed (see a more detailed explanation below), which could be undermining the main conclusions of the study. At the moment some of the conclusions need to be supported by additional analysis, more explicit presentation of the results and improved comparison to the competing methods.

**Are sufficient details provided to allow replication and comparison with related analyses that may have been performed?**

Yes, however the results of the comparison should be better highlighted in additional display items.

**Is the method likely to be of broad utility? Is any software component easy to install and use?**

Rahmani et al propose a novel semi-supervised method for improving the deconvolution by using prior knowledge on the distribution of cell type proportions. A successful incorporation of this type of prior information has not been reported so far. The authors embark upon Bayesian modelling approach, and implement it using an efficient algorithmic scheme. The implementation of the method is published on a popular code repository and can be applied by the majority of potential users.

**Is the paper of broad interest to others in the field, or of outstanding interest to a broad audience of biologists?**

Yes: The problem of deconvolution applied to high-dimensional DNA methylation data has become seminal in bioinformatics and computational epigenetics literature. Given largely prohibitive cost of obtaining clean cell populations, it has direct implications for the steadily growing field of epigenetic association studies. Furthermore, due to favourable quantitative properties of DNA methylomes the problem has turned into a playground where advanced computational methods can be tested, making it interesting even to a broader audience of statisticians and machine learning researchers. Given that the available methods are still short of providing an ultimate solution for this problem, any new methodological advance is highly relevant for the respective fields and research communities.

Comments to author:

--- Naturally for a semi-supervised method, one potential danger for the user when applying BayesCCE lies in an erroneously estimated prior information. For instance, this can be especially deteriorating in a very widespread case of cell type proportions being different between cases in controls in a typical two-group EWAS. A failure to detect this difference early is expected to bias the complete analysis, while a reference-based method would be able to deal with such a case. A related situation is when the prior is not informative, i.e. the estimated Dirichlet parameters are not significantly different from each other. The authors should more extensively discuss this issue and demonstrate the effects of a wrongly estimated and uninformative prior upon the performance of BayesCCE.

--- Many conclusions of the BayesCCE are implicitly based upon the assumption that a typical morphologically and immunochemically defined cell type is characterized by a stable DNA methylation signature that is globally separable from those of other cell types present in the measured mixture. This is a rather strong assumption even for the relatively well studied case of hematopoietic cell types. It is sufficient to look at PCA of the most popular reference data set (Reinius et al, Figure 1b) to see that globally one can relatively easily separate myeloid and lympoid lineages as well as the B-cell compartment, while the finer cell types and subtypes are largely overlapping. Moreover, mounting evidence from whole-genome studies (Kullis et al, Nat Gen, 2016; Oakes et al, Nat Gen, 2016; Durek et al, Immunity, 2016) implies that, at least in lymphocytes, changes of DNA methylation attributable to the transition from naïve to memory state might be much larger than those specific to canonical subtypes. If this is the case, reference-free methods are bound to recover different signatures as those obtained by the canonical surface marker-based cell isolation. As a result, reference-based and reference-free proportion estimates will be hardly comparable. The authors should either rule out such a possibility by their own or published analysis, or discuss it in the context of their benchmark.

---- Elaborating on the previous point, it is somewhat unfortunate that the authors concentrate upon the recovery of cell type proportions and fully omit any analysis of deconvoluted cell type methylomes which are equally obtainable by BayesCCE (matrix M). While good correlation of the recovered and ground truth proportions indirectly implies good recovery of the cell type methylomes, it would be of small additional effort, yet very important to clearly demonstrate this. This can be done via correlation matrices, clustering or by any other similar approach.

---- Consequently, a recurrent claim in the manuscript is that in contrast to NNMF and MeDeCom, which return components representing linear combinations of the cell type methylomes, BayesCCE components show more direct correspondence to pure cell types. Nevertheless, even disregarding the problem of cell type definitions, this is not directly shown in any of the display items. A supplementary figure or table with all correlations supporting the values reported in Figure 1 would greatly help to convey and substantiate the claim of improved identifiability.

--- Even assuming nice hierarchical separability of the canonical cell type methylomes, the comparison to reference-free methods is not completely fair in two respects:

(1) The number of components k has a natural upper bound in case of BayesCCE, and is limited by the number of cell types with available prior information, k'. In case of reference-free methods the only hard upper bound is the number of columns of the input methylation matrix. While both NNMF and MeDeCom suggest methods for selecting a reasonable k, in their comparison authors seem to use a fixed k=k' in both cases. In the same time, it appears that BayesCCE is implicitly exploring a larger space of potential components through d>k' ReFACTor PCs. For a fair comparison, k selection should be applied for both reference-free scenarios. In the most likely case of k>k' thereby obtained components can then be selected or aggregated based on their similarity to the k' references.

(2) BayesCCE has achieved the largest advance over reference-free methods in the "imputation" and "extension" cases where samples with known proportions were added. While in general it is not surprising that a semi-supervised method performs better than fully unsupervised ones, another, more comparable semi-supervised scenario for case of the reference-free methods would be to extend the target data with available reference methylomes. Note that this is essentially the same additional information as when mixed samples with known cell type proportions used, since reference methylomes can be easily inferred from the latter (a sub-problem with fixed R). Importantly, reference-free methods cannot be explicitly instructed with regard to which columns of the input matrix contain the cell type methylomes, making this scenario principally distinct from reference-based estimation. Even though this would not completely fit into the focus of the study and to the narrative of reference methylome being hardly obtainable, such analysis might help in resolving the earlier raised issues.

--- In their non-identifiability proof the authors nicely adapt the well-known non-uniqueness of NNMF solutions to the concrete example of DNA methylation data. It is indeed a potentially valid explanation behind the NNMF and related methods not being able to recover the expected cell type methylomes and proportions. In fact, this can be relatively easily verified. The proposition implies that true M' and R' (assumed by the authors to be sufficiently close to the reference methylomes and their proportions estimated by reference-based methods) comprise one of many optimal solutions to (6) which NNMF schemes fail to identify. If this is the case, a simple NNMF scheme, initialized with M' and R', should quickly converge, returning the deconvolution products very similar to the initial values. In the opposite case, a more optimal solution to (6) exists than the assumed one.

--- In this respect, it would also be very helpful if, along with MAC and MAE of the cell type proportions, authors also reported the approximation error of (6) for all the analysis runs performed.

--- Most of the presented analysis examples are performed on large data sets where cell counts are not available. However, in their earlier published study (Rahmani et al., Nat Methods, 2016) a large data set was analyzed where cell counts were available for a large portion of the samples (denoted GALA II in the original paper). It is surprising that the authors decided to omit this data set, as it could have been a great resource to demonstrate the advantages of BayesCCE.

--- Finally, the authors extensively demonstrate the applicability of their method to cell type discovery in whole blood. It is true that blood-based EWAS are indeed the most widespread application scenario, but nevertheless reference methylomes of blood cell types are largely accessible and reference-based methods seem to do a decent job. In the same time, they benchmark against reference-free deconvolution methods which are expected to be more useful in more complex scenarios with scarcely available reference methylomes and much less general knowledge about the cell mixture composition. An alternative biological scenario of such kind would be helpful to demonstrate the benefits of BayesCCE.

Minor points:

--- p. 26, l.41 "a cell counts"

--- legend of Fig. S4: "six constituting cell types in blood (k = 3)"

**Reviewer 2**

**Are the methods appropriate to the aims of the study, are they well described, and are necessary controls included?**
Yes methods are appropriate. Some additional scenarios should be considered. see detailed comments.

**Are the conclusions adequately supported by the data shown?**

yes

**Are sufficient details provided to allow replication and comparison with related analyses that may have been performed?**

yes

**Is the method likely to be of broad utility? Is any software component easy to install and use?**

Moderate utility

**Is the paper of broad interest to others in the field, or of outstanding interest to a broad audience of biologists?**

No

Comments to author:

The paper by Rahmani et al. describes an empirical Bayes approach to estimated cell type proportions in DNA methylation data. The estimation of correct cell type proportions is well demonstrated with the new method, and the ability to incorporate cell count data on a subset of the samples is very appealing. The non-identifiability question is well explained. Since BayesCCE depends on the use of informative priors, it could be said to some extent that improve performance could be expected.

1.	The paper does not sufficiently address an important point, which is whether differences in cell type proportions associated with a covariate (X) are biased or unbiased when using BayesCCE in comparison to other methods. The discussion says "In case merely a correction is desired, we suggest …" which could be interpreted as referring to inference with respect to a covariate X., However this single sentence seems inadequate. Suppose cell type proportions are quite different between two groups (cases/controls) or vary importantly with a continuous covariate. Surely this is of interest. Does BayesCCE pull the estimates towards a common middle ground (i.e. similar to the empirical prior)? If you have a few cell counts across the range of X, does any such bias towards the null largely disappear? In particular, if there is a situation with confounding such that X is associated both with a change in cell type composition and with changing methylation levels (at some sites), is it a good idea to use BayesCCE?

2.	More investigation should be done into performance under the misspecification of the assumed number of cell types K. Simulation results were obtained when assuming K=3, but what happens when the assumed number of cell types is larger than the actual number present in the sample (or at least the true proportions of certain cell types in the samples are very small)?

3.	The authors made a point to show the non-identifiability of the non-negative matrix factorization (NNMF) model. Even though the estimates of the model with the Dirichlet prior tend to be drawn more towards the true cell type proportions than in the NNMF model, is this new model now identifiable? Could this be clearer in the manuscript?

4.	The issues of precision are brought into Methods (relaxing exact constraints in various ways) but are not given adequate discussion. Why is this needed? What kind of inaccuracy could this lead to? Would you expect biases? What additional data would be needed such that these relaxations would not be required, if such a possibility could be imagined? Does it affect their proofs? Does it affect identifiability? Should sensitivity analyses be performed?

Minor points:

Since the article claims to be able to directly estimate cell proportions rather than just linear combinations, then the results should be more focused on absolute errors rather than correlations (i.e. reverse the rows in Figures such as Figure 2). Moreover, errors of each estimated cell type should be presented in addition to the errors averaged over all cell types. For example, in Figure 4 the mean absolute error appears to flatten out after about 20 samples with known cell counts, but is this error simply dominated by the most prevalent cell type?

We identified some minor grammatical or typographical errors:

"heterogeneous source consisted of …" line 10 page 14. Should be "consisting"

Page 26, line 41: "where a cell counts are known"

Page 16 line 14 : perhaps "non-identifiable nature"?

Page 18 line 23: "retrun"

Page 22 line 56: "rates"

Page 2 line 43 - "ubiquitous"

Page 13 line 24 - "return"

**Authors’ Response**

Reviewer #1:

--- Naturally for a semi-supervised method, one potential danger for the user when applying BayesCCE lies in an erroneously estimated prior information. For instance, this can be especially deteriorating in a very widespread case of cell type proportions being different between cases in controls in a typical two-group EWAS. A failure to detect this difference early is expected to bias the complete analysis, while a reference-based method would be able to deal with such a case. A related situation is when the prior is not informative, i.e. the estimated Dirichlet parameters are not significantly different from each other. The authors should more extensively discuss this issue and demonstrate the effects of a wrongly estimated and uninformative prior upon the performance of BayesCCE.

*We thank the reviewer for raising and emphasizing this important issue. We added an entirely new subsection including a set of new experiments to address and discuss the issue of potential biases introduced into BayesCCE by the prior information (see “Robustness of BayesCCE to biases introduced by the cell composition prior” under the Results section). Through a series of extensive experiments, we found BayesCCE to handle such biases remarkably well. Specifically, we included the following additional analyses:*

-	We verified that cell counts estimated by BayesCCE capture differences between populations (e.g., cases versus controls) even when using a single general prior to describe all the individuals in the data (Figure 5). Our new experiments demonstrate that BayesCCE leverages the prior information in a way that directs the optimization towards estimation of cell counts, while not imposing too strong constraints that would lead to missing natural cell composition variability between different populations (such as variability between cases and controls).

-	We evaluated the performance of BayesCCE under different levels of noise added to the prior information (Supplementary Figure S9). As expected, we found very noisy priors to impact the performance of BayesCCE, however, even in the presence of a non-informative prior, BayesCCE overall outperformed existing reference-free methods.

-	We updated our implementation to allow incorporation of more than one prior into BayesCCE. Yet, we show that incorporating two priors (e.g., a separate prior for cases and for controls) provides no improvement compared with using a single general prior (Supplementary Table T3). This further confirms our conclusion that BayesCCE handles the bias introduced by using a single prior remarkably well.

*We further added an appropriate discussion about the above issues under the Discussion section.*

--- Many conclusions of the BayesCCE are implicitly based upon the assumption that a typical morphologically and immunochemically defined cell type is characterized by a stable DNA methylation signature that is globally separable from those of other cell types present in the measured mixture. This is a rather strong assumption even for the relatively well studied case of hematopoietic cell types. It is sufficient to look at PCA of the most popular reference data set (Reinius et al, Figure 1b) to see that globally one can relatively easily separate myeloid and lympoid lineages as well as the B-cell compartment, while the finer cell types and subtypes are largely overlapping. Moreover, mounting evidence from whole-genome studies (Kullis et al, Nat Gen, 2016; Oakes et al, Nat Gen, 2016; Durek et al, Immunity, 2016) implies that, at least in lymphocytes, changes of DNA methylation attributable to the transition from naïve to memory state might be much larger than those specific to canonical subtypes. If this is the case, reference-free methods are bound to recover different signatures as those obtained by the canonical surface marker-based cell isolation. As a result, reference-based and reference-free proportion estimates will be hardly comparable. The authors should either rule out such a possibility by their own or published analysis, or discuss it in the context of their benchmark.

*Indeed, cell types which are not characterized by stable DNA methylation signatures that are globally separable from those of other cell types in the input data are not expected to be estimated well. This problem is clearly not unique to BayesCCE but general to any reference-free or reference-based method. For example, the reference-based method looks for distinct cell-type specific methylomes which can be used for a decomposition, as considering non-distinct methylomes (or, equivalently, cell types with no distinct signatures) will result in a non-identifiable problem. Yet, it is widely recognized that the analysis of methylation data requires a correction for cell type composition (Jaffe & Irrizari, 2012), and thus these methods are widely used despite their inherent limitation presented by the reviewer. Bounded by this clear inherent limitation of all methods for estimating cell type composition, we believe that the second argument made by the reviewer regarding the strength of signals is of more interest.*

*In general, reference-free methods do not guarantee to disentangle signals of interest from other background signals. In the context of estimating cell type composition from DNA methylation, while a reference-free approach has proved useful in many cases, in scenarios where the variability of some particular cell type is weaker than the variability of other sources of variation, reference-free methods are expected to provide limited accuracy for estimating that cell type. This problem is obviously not unique to BayesCCE, but also applies to any reference-free method which does not leverage external reference data (in this context, we do not regard the cell composition distribution prior used by BayesCCE as a reference data). We note that, at least to some extent, this problem is expected to also affect reference-based methods, as cell types with weaker signatures are expected to lead to a more noisy decomposition. This, in fact, may provide an explanation for the relatively poor performance of the reference-based method in estimating monocyte proportions (Yousefi et al., Environmental and molecular mutagenesis 2015, Cardenas et al., Epigenetics 2016, Rahmani et al., Nature Methods 2016).*

*We added the following discussion to the paper (under the Discussion section):*

*“Since BayesCCE relies on the ReFACTor components, it is limited by their quality, and particularly, if the variability of some cell type is not captured by ReFACTor, BayesCCE will not be able to estimate that cell type well. Such a result is possible in scenarios where the variation of a particular cell type is substantially weaker than other sources of variation in the data (which are unrelated to cell type composition); we note, however, that this potential limitation is not exclusive for ReFACTor or BayesCCE but rather a general limitation of all existing reference-free methods.”*

---- Elaborating on the previous point, it is somewhat unfortunate that the authors concentrate upon the recovery of cell type proportions and fully omit any analysis of deconvoluted cell type methylomes which are equally obtainable by BayesCCE (matrix M). While good correlation of the recovered and ground truth proportions indirectly implies good recovery of the cell type methylomes, it would be of small additional effort, yet very important to clearly demonstrate this. This can be done via correlation matrices, clustering or by any other similar approach.

*We added a new paragraph and an additional experiment in order to address and discuss this alternative approach for evaluating the performance of BayesCCE (See the last paragraph under the subsection titled “BayesCCE impute: cell counts imputation” in the Results section). Particularly, per the reviewer’s suggestion, we added a new figure which demonstrates the recovery of cell-type specific methylation profiles by BayesCCE (Supplementary Figure S8).*

---- Consequently, a recurrent claim in the manuscript is that in contrast to NNMF and MeDeCom, which return components representing linear combinations of the cell type methylomes, BayesCCE components show more direct correspondence to pure cell types. Nevertheless, even disregarding the problem of cell type definitions, this is not directly shown in any of the display items. A supplementary figure or table with all correlations supporting the values reported in Figure 1 would greatly help to convey and substantiate the claim of improved identifiability.

*We included two supplementary tables to fully describe the performance of each method in recovering the composition of each cell type by each one of the methods we benchmarked (Supplementary Tables T1 and T2).*

--- Even assuming nice hierarchical separability of the canonical cell type methylomes, the comparison to reference-free methods is not completely fair in two respects:

(1) The number of components k has a natural upper bound in case of BayesCCE, and is limited by the number of cell types with available prior information, k'. In case of reference-free methods the only hard upper bound is the number of columns of the input methylation matrix. While both NNMF and MeDeCom suggest methods for selecting a reasonable k, in their comparison authors seem to use a fixed k=k' in both cases. In the same time, it appears that BayesCCE is implicitly exploring a larger space of potential components through d>k' ReFACTor PCs. For a fair comparison, k selection should be applied for both reference-free scenarios. In the most likely case of k>k' thereby obtained components can then be selected or aggregated based on their similarity to the k' references.

*We note that in BayeCCE, as well as in all other dimensionality reduction algorithms such as NNMF and MeDeCom, there is a distinction between the number of dimension that the algorithm emits (k), and the number of dimension that it explores. Particularly, if the original rank of the matrix is r, then all these methods provide a k-dimensional output by exploring the entire input space of dimension r > k. In other words, our use of d > k’ ReFACTor PCs is still a valid and fair comparison to the other methods since these PCs are only used in the exploration stage, and we eventually output k dimensions using BayesCCE. The number of dimensions in the output is what counts, and not the number of dimensions explored by the algorithm. By allowing each method to output data with the same dimension, as we do, we allow a fair comparison in our evaluation of the different methods.*

*We agree that considering only k components for the reference-free methods may restrict them from capturing variation of interest. However, the focus of this paper is the scenario wherein we are interested in cell count estimates for a predefined set of cell types. Therefore, outputting a larger number of components than the number of preselected cell types would pose a serious interpretability issue. Particularly, it is not clear how this approach can be useful in real world settings, as our problem definition assumes that appropriate reference data is unavailable and selection or aggregation of components would require some sort of reference data. Moreover, we would like to note that estimating the dimension of a variation of interest in data (e.g., number of cell types) is generally an open problem. We are aware of the methods that were previously suggested for selecting the number of cell types k, however, these methods implicitly assume that the entire signal in the data consists of cell type composition variation, an assumption which clearly does not hold in reality, given the many other factors that are known to substantially contribute to variation in methylation data (e.g., age, gender, smoking, genetics, batch effects etc.).*

(2) BayesCCE has achieved the largest advance over reference-free methods in the "imputation" and "extension" cases where samples with known proportions were added. While in general it is not surprising that a semi-supervised method performs better than fully unsupervised ones, another, more comparable semi-supervised scenario for case of the reference-free methods would be to extend the target data with available reference methylomes. Note that this is essentially the same additional information as when mixed samples with known cell type proportions used, since reference methylomes can be easily inferred from the latter (a sub-problem with fixed R). Importantly, reference-free methods cannot be explicitly instructed with regard to which columns of the input matrix contain the cell type methylomes, making this scenario principally distinct from reference-based estimation. Even though this would not completely fit into the focus of the study and to the narrative of reference methylome being hardly obtainable, such analysis might help in resolving the earlier raised issues.

*We agree with the reviewer that investigating the potential advantages of combining existing unsupervised approaches with reference methylomes is of interest. However, it is in fact the case that reference methylomes are hardly obtainable. This is probably best reflected by the fact that even though hundreds of whole-blood methylation studies have been conducted in the last few years, the go-to blood reference is still a fairly small one (n=6), which is not expected to represent every population well. This has been highlighted in a recent example with a newborn population (Yousefi et al., Environmental and molecular mutagenesis 2015). For that reason, our focus in this study is estimation of cell-type proportions without the need for methylation reference, a task which so far has not been shown to be robustly possible in real data by any method.*

*Moreover, we would like to note that while incorporating reference methylomes may in principle provide the same amount of information to the problem as incorporating cell counts, the latter approach is expected to be more useful in practice. Cell counts for a small fraction of the samples in a study are expected to be relatively easily obtainable, and leveraging such data is expected to allow a reasonable recovery of the cell-type-specific methylomes of the specific population in the study (matrix M). On the other hand, since obtaining reference methylomes is harder in practice (it requires cell sorting, as well as measuring the methylation levels in each cell type, which is prohibitively expensive for large samples), for most studies it would be much more reasonable to consider previously collected methylomes, which will not necessarily allow to recover important population-specific variability of the methylomes.*

*Finally, as implied by the reviewer, reference methylomes can be leveraged in different ways (i.e. not just the approach taken by the popular reference-based method by Houseman et al.). Yet, given such information makes the inference problem to be reference-based, as the solution depends upon the reference methylomes, rather than reference-free or semi-supervised.*

*Due to the above reasons, we feel that investigating the possibility of incorporating reference methylomes is out of the scope of this paper..*

--- In their non-identifiability proof the authors nicely adapt the well-known non-uniqueness of NNMF solutions to the concrete example of DNA methylation data. It is indeed a potentially valid explanation behind the NNMF and related methods not being able to recover the expected cell type methylomes and proportions. In fact, this can be relatively easily verified. The proposition implies that true M' and R' (assumed by the authors to be sufficiently close to the reference methylomes and their proportions estimated by reference-based methods) comprise one of many optimal solutions to (6) which NNMF schemes fail to identify. If this is the case, a simple NNMF scheme, initialized with M' and R', should quickly converge, returning the deconvolution products very similar to the initial values. In the opposite case, a more optimal solution to (6) exists than the assumed one.

*Given an optimal solution, we do not understand what would it mean to get “a more optimal solution”. We kindly ask the reviewer to clarify their comment.*

--- In this respect, it would also be very helpful if, along with MAC and MAE of the cell type proportions, authors also reported the approximation error of (6) for all the analysis runs performed.

*Given the non-identifiability nature of this problem, the approximation error of (6) is not very informative with respect to the quality of the cell count estimates. Particularly, our proof suggests that an NNMF solution with a better approximation error does not necessarily mean better cell composition estimates. Furthermore, a typical optimal solution of BayesCCE (and of MeDeCom), which takes a form of a penalized NNMF, is expected to provide worse approximation error compared with an optimal solution of a standard NNMF objective. Yet, as we show in the paper through an extensive set of experiments, BayesCCE outperform the alternatives in terms of both correlation and absolute error. This can be explained in part by the fact that the approximation error in (6) may require using sources of variation other than cell type composition in order to be minimized, and therefore a standard NNMF solution would pick such signals.*

--- Most of the presented analysis examples are performed on large data sets where cell counts are not available. However, in their earlier published study (Rahmani et al., Nat Methods, 2016) a large data set was analyzed where cell counts were available for a large portion of the samples (denoted GALA II in the original paper). It is surprising that the authors decided to omit this data set, as it could have been a great resource to demonstrate the advantages of BayesCCE.

*Indeed, we have previously used the GALA II data set (Rahmani et al., Nature Methods 2016), in which cell counts are available for a subset of the individuals in the data. However, these cell counts were measured in a few months difference from the methylation levels, resulting in a lower accuracy of the cell count estimates by all methods. It has recently been suggested that due to this issue, the GALA II data is not ideal for benchmarking of methods for estimating cell composition (Zheng et al., Nature methods 2017, Rahmani et al., Nature methods 2017), and we therefore decided to omit this data set from our evaluation of BayesCCE, as we feel that the evidence is compelling without adding this dataset. We note that we did evaluate the performance of BayesCCE and the other reference-free methods using the GALA II data set as well, and we found BayesCCE to overall outperform the alternative methods. Specifically, considering the measured cell counts as the ground truth (k=3; only counts of granulocytes, lymphocytes and monocytes were available), we found the best performing method to be BayesCCE imp (MAC 0.46, MAE 0.015). The best performance among the alternative reference-free methods was MAC 0.381 and MAE 0.261. We further found BayesCCE imp to perform best when considering the reference-based estimates as the ground truth, with MAC 0.84 and MAE 0.015 for k=3, and MAC 0.62 and MAE 0.03 for k=6, as opposed to the best performance among the alternative reference-free methods: MAC 0.58 and MAE 0.11 for k=3, and MAC 0.37 and MAE 0.14 for k=6.*

--- Finally, the authors extensively demonstrate the applicability of their method to cell type discovery in whole blood. It is true that blood-based EWAS are indeed the most widespread application scenario, but nevertheless reference methylomes of blood cell types are largely accessible and reference-based methods seem to do a decent job. In the same time, they benchmark against reference-free deconvolution methods which are expected to be more useful in more complex scenarios with scarcely available reference methylomes and much less general knowledge about the cell mixture composition. An alternative biological scenario of such kind would be helpful to demonstrate the benefits of BayesCCE.

*We agree with the reviewer that looking at biological scenarios other than whole-blood is of interest. Unfortunately, it is hard to evaluate more complex scenarios with the currently available data. Specifically, in order to evaluate BayesCCE on data from other tissues, one would need a ground truth cell-counts for the studied tissue or alternatively a reference data that has been shown to provide a good surrogate for the ground truth. To the best of our knowledge, such data is not available except for blood tissue. We therefore opt to focus on evaluating the performance of BayesCCE using multiple large whole-blood data sets - as the reviewer pointed out, the current reference data for whole-blood tissue is now acknowledged to do a decent job in estimating cell counts, and it therefore provides an opportunity to conduct a reliable benchmarking.*

*We would like to note that beyond its potential utility for complex biological scenarios for which reference is unavailable, BayesCCE may also provide an opportunity to improve cell count estimates in whole-blood studies in scenarios where the currently available reference data is not appropriate. Notably, in a recent work we have shown that ReFACTor outperforms the reference-based method in correction for cell composition in multiple whole-blood data sets (Rahmani et al., Nature Methods 2017). Differences in performance between ReFACTor (upon which BayesCCE relies for obtaining a starting point which captures the cell composition variation in the data) and the reference-based method are expected to be especially large in studies where the available reference data do not represent the individuals in the study well. We argue that this is likely to be typically the case, as the current go-to whole blood reference consists of only six individuals, which represent a very specific and narrow population in terms of methylome altering factors, such as genetics, age and gender. That said, large data sets with cell counts are required in order to fully investigate and demonstrate these claims.*

*In order to clarify these issues, we added a new paragraph to the paper (see the second to last paragraph under the Discussion section).*

Minor points:

--- p. 26, l.41 "a cell counts"

--- legend of Fig. S4: "six constituting cell types in blood (k = 3)"

*We thank the reviewer for pointing out these errors.*

**Reviewer 2:**

The paper by Rahmani et al. describes an empirical Bayes approach to estimated cell type proportions in DNA methylation data. The estimation of correct cell type proportions is well demonstrated with the new method, and the ability to incorporate cell count data on a subset of the samples is very appealing. The non-identifiability question is well explained. Since BayesCCE depends on the use of informative priors, it could be said to some extent that improve performance could be expected.

1. The paper does not sufficiently address an important point, which is whether differences in cell type proportions associated with a covariate (X) are biased or unbiased when using BayesCCE in comparison to other methods. The discussion says "In case merely a correction is desired, we suggest …" which could be interpreted as referring to inference with respect to a covariate X., However this single sentence seems inadequate. Suppose cell type proportions are quite different between two groups (cases/controls) or vary importantly with a continuous covariate. Surely this is of interest. Does BayesCCE pull the estimates towards a common middle ground (i.e. similar to the empirical prior)? If you have a few cell counts across the range of X, does any such bias towards the null largely disappear? In particular, if there is a situation with confounding such that X is associated both with a change in cell type composition and with changing methylation levels (at some sites),

is it a good idea to use BayesCCE?

*We thank the reviewer for raising and emphasizing this important issue. Please see our comment to Reviewer #1 on this matter, where we describe additional analyses we performed to address these questions. Our additional analyses show that BayesCCE handles remarkably well the biases introduced by including a prior which does not necessarily represent well all the samples in the data. Particularly, we show that for both the case of a binary covariate affecting the cell composition distribution (case/control status) and the case of a quantitative phenotype (age), BayesCCE robustly provides estimates which reflect the expected variability with respect to the covariate affecting the cell composition (rather than pulls the estimates towards one shared distribution). For more details, please see the subsection titled “Robustness of BayesCCE to biases introduced by the cell composition prior” under the Results section.*

*Beyond the robustness of BayesCCE to covariates affecting the cell composition distribution, we agree that it is important to account for covariates which may affect the methylation levels. We note that BayesCCE does provide a way to handle covariates which are expected to affect methylation levels (see the last paragraph under the “Algorithm” subsection in the Methods section). However, factors affecting the methylation levels may also affect the performance of ReFACTor, which in turn can affect BayesCCE which relies upon the ReFACTor output as a starting point. It is therefore important to follow the guidelines for the application of ReFACTor. We added the following sentence to the Discussion section:*

*“It is therefore important to follow the guidelines for the application of ReFACTor such as incorporation of methylation altering covariates; these guidelines we recently highlighted elsewhere.”*

*Finally, we note that the referred sentence from the Discussion section (“In case merely a correction is desired...”) did not concern the case of the cell type composition being correlated with covariates, but rather it discussed the case of correcting for cell type composition in EWAS. We rewrote this sentence to improve clarity.*

2. More investigation should be done into performance under the misspecification of the assumed number of cell types K. Simulation results were obtained when assuming K=3, but what happens when the assumed number of cell types is larger than the actual number present in the sample (or at least the true proportions of certain cell types in the samples are very small)?

*The prior information is expected to encode the knowledge about the studied tissue, and therefore there is no reason to include prior information on cell types which are not known to exist in the sample. Regarding the second question about lowly abundant cell types that was raised by the reviewer, our experiments throughout the paper clearly show that BayesCCE in fact provides better performance in capturing the variation of more abundant cell types compared with less abundant cell types. Of course, this is a general problem which is not unique to BayesCCE but also applies to any reference-free method, owing to the fact the lowly abundant cell types contribute less to the total signal composing the heterogeneous input data.*

3. The authors made a point to show the non-identifiability of the non-negative matrix factorization (NNMF) model. Even though the estimates of the model with the Dirichlet prior tend to be drawn more towards the true cell type proportions than in the NNMF model, is this new model now identifiable? Could this be clearer in the manuscript?

*Obviously, if the weight of the prior is large enough (which would be reflected in our case with very high values of the Dirichlet parameters), the solution of BayesCCE will be identifiable (yet uninteresting). It is therefore a question of which conditions should the prior satisfy (or what level of constraint should be introduced into the analysis) in order for BayesCCE to provide a unique solution. We currently do not have a proof for such conditions. To clarify this, we added the following sentences under the Discussion section:*

*“We show mathematically and empirically the non-identifiability nature of the more straightforward reference-free NNMF approach for inferring cell counts, which tends to provide only linear combinations of the cell counts. In contrast, while we do not provide conditions for the uniqueness of a BayesCCE solution, our empirical evidence from multiple data sets clearly demonstrates the success of BayesCCE in providing desirable results of one component per cell type by leveraging readily obtainable priors from previously collected cell counts.”*

4. The issues of precision are brought into Methods (relaxing exact constraints in various ways) but are not given adequate discussion. Why is this needed? What kind of inaccuracy could this lead to? Would you expect biases? What additional data would be needed such that these relaxations would not be required, if such a possibility could be imagined? Does it affect their proofs? Does it affect identifiability? Should sensitivity analyses be performed?

*We first note that these relaxations do not affect our non-identifiability proof, as these relaxations only add degrees of freedom to a system which is already non-identifiable in the first place.*

*We discuss three different relaxations of constraints in the Methods section under the subsection “Implementation and practical issues”:*

*-	We require the estimated cell type proportions to be greater than 0 (rather than greater or equal to 0) by requiring them to be greater than an arbitrarily small value (10e-4).*

*-	We allow a small deviation from the constraint which requires the sum of cell proportions of each sample to be 1.*

*-	In the case where cell counts are known for a subset of the samples in the study, we allow for the linear transformations of the samples with known cell counts to result in cell type proportions which are slightly different than the input cell counts.*

*The first constraint is essential for the optimization in order to avoid invalid evaluations of the objective function (and therefore a subsequent failure in the entire optimization) because the logarithm term in the objective function cannot take non-positive values.*

*The last two relaxations are important in order to assure feasibility of the BayesCCE solution. In order to see that, consider the standard NNMF formulation in (6)-(9). Given an estimate of M we can easily estimate R (a reference-based problem) and vice versa. A typical solution to the NNMF problem alternates between these two sub-problems. This means that in each step we solve a set of several independent problems - given M, we learn each row of R separately (i.e. the cell composition of each individual), and given R, we learn each row of M separately (i.e. the cell-type specific mean methylation levels of each CpG site). For each such sub-problem, assuming the fixed variables satisfy the constraints, it is easy to see that there is necessarily a feasible solution in spite of the constraints in (7)-(9). In contrast, in BayesCCE, where we fit M and R jointly, we learn linear transformations which are required to satisfy the constraints for all of the rows in R and all of the rows in M jointly in one step. Since the starting point of BayesCCE is essentially a set of principal components (the ReFACTor components), which do not necessarily capture only cell composition variability, precisely satisfying the constraints in (7)-(9) is expected to not always be possible. We verified this empirically and therefore added the above relaxations in order to allow feasible solutions. Importantly, we observed that these relaxations eventually result in feasible solutions which typically tend to tightly concentrate around the original constraints.*

*We rewrote the subsection “Implementation and practical issues” under the Methods section in order to clarify these issues.*

Minor points:

Since the article claims to be able to directly estimate cell proportions rather than just linear combinations, then the results should be more focused on absolute errors rather than correlations (i.e. reverse the rows in Figures such as Figure 2). Moreover, errors of each estimated cell type should be presented in addition to the errors averaged over all cell types. For example, in Figure 4 the mean absolute error appears to flatten out after about 20 samples with known cell counts, but is this error simply dominated by the most prevalent cell type?

BayesCCE aims at providing a set of components such that each component captures one cell type. This, in general, may result in bad absolute errors. As we show, incorporating cell counts for a small subset of the samples in the study leads BayesCCE to capture calibrated estimates which result in low absolute errors (and a further increase in correlation). However, since we anticipate BayesCCE to be mostly applied in the context of analyses which require high correlation but not necessarily estimates that are calibrated in terms of absolute values, we opt to visually emphasize the correlation quality measurement in some of the figures. Of course, for all experiments, we equally report both of these quality measurements.

*We included a summary of the performance of all methods we benchmarked while providing a break down into individual cell types (Supplementary Tables T1 and T2).*

We identified some minor grammatical or typographical errors:

"heterogeneous source consisted of …" line 10 page 14. Should be "consisting"

Page 26, line 41: "where a cell counts are known"

Page 16 line 14 : perhaps "non-identifiable nature"?

Page 18 line 23: "retrun"

Page 22 line 56: "rates"

Page 2 line 43 - "ubiquitous"

Page 13 line 24 - "return"

*We thank the reviewer for pointing out these errors.*

**Second round of review**

**Reviewer 1:**

AU: We thank the reviewer for raising and emphasizing this important issue. We added an entirely new subsection including a set of new experiments to address and discuss the issue of potential biases introduced into BayesCCE by the prior information (see "Robustness of BayesCCE to biases introduced by the cell composition prior" under the Results section). Through a series of extensive experiments, we found BayesCCE to handle such biases remarkably well. Specifically, we included the following additional analyses:

- We verified that cell counts estimated by BayesCCE capture differences between populations (e.g., cases versus controls) even when using a single general prior to describe all the individuals in the data (Figure 5). Our new experiments demonstrate that BayesCCE leverages the prior information in a way that directs the optimization towards estimation of cell counts, while not imposing too strong constraints that would lead to missing natural cell composition variability between different populations (such as variability between cases and controls).

- We evaluated the performance of BayesCCE under different levels of noise added to the prior information (Supplementary Figure S9). As expected, we found very noisy priors to impact the performance of BayesCCE, however, even in the presence of a non-informative prior, BayesCCE overall outperformed existing reference-free methods.

- We updated our implementation to allow incorporation of more than one prior into BayesCCE. Yet, we show that incorporating two priors (e.g., a separate prior for cases and for controls) provides no improvement compared with using a single general prior (Supplementary Table T3). This further confirms our conclusion that BayesCCE handles the bias introduced by using a single prior remarkably well.

We further added an appropriate discussion about the above issues under the Discussion section.

R1: The experiments look convincing, good job, although it seems that proportions returned by BayesCCE can sometimes contain false-positive associations (see e.g. NK cells by basic BayesCCE in Hannum data, Monocytes by BayesCCE imp in Hannon II etc ) while also having false-negatives (Monocytes by BayesCCE imp ext in Hannum, NK cells by BayesCCE im in Hannon I etc ).

------

AU: Indeed, cell types which are not characterized by stable DNA methylation signatures that are globally separable from those of other cell types in the input data are not expected to be estimated well. This problem is clearly not unique to BayesCCE but general to any reference-free or reference-based method. For example, the reference-based method looks for distinct cell-type specific methylomes which can be used for a decomposition, as considering non-distinct methylomes (or, equivalently, cell types with no distinct signatures) will result in a non-identifiable problem. Yet, it is widely recognized that the analysis of methylation data requires a correction for cell type composition (Jaffe & Irrizari, 2012), and thus these methods are widely used despite their inherent limitation presented by the reviewer. Bounded by this clear inherent limitation of all methods for estimating cell type composition, we believe that the second argument made by the reviewer regarding the strength of signals is of more interest.

< omitted part of the response to fit into the character limit>

We added the following discussion to the paper (under the Discussion section):

"Since BayesCCE relies on the ReFACTor components, it is limited by their quality, and particularly, if the variability of some cell type is not captured by ReFACTor, BayesCCE will not be able to estimate that cell type well. Such a result is possible in scenarios where the variation of a particular cell type is substantially weaker than other sources of variation in the data (which are unrelated to cell type composition); we note, however, that this potential limitation is not exclusive for ReFACTor or BayesCCE but rather a general limitation of all existing reference-free methods."

R1: Unfortunately, the main message of my initial comment did not get through, perhaps due to an insufficiently clear formulation. The point is that the classical cell types, usually defined via various cell surface markers, do not necessarily correspond to cell types which one would identify given sufficient knowledge about the epigenomes of all cells present in a certain tissue. Moreover, proportions and methylomes of the latter cell types will most likely appear as linear combinations of the former and vice versa. In that respect the existing reference-based methods are by design limited to recover only the proportions of surface marker-defined cell types. Formulated more directly, reference-based methods with references such as Reinius et al. (as well as analogous cell counting approaches) predict nothing but proportions of cells baring a certain surface marker. Reference-free methods have, at least, some chances to detect the true "epigenetic" cell types. A strict benchmark of the kind the authors have performed is, therefore, only feasible iff they can show that these two cell type definitions are at least roughly equivalent. Otherwise, the lack of correspondence between the results of reference-based and reference-free methods as central argument for BayesCCE is rather weak. In any case, these issues have to be extensively covered in the Discussion.

-----

AU: We added a new paragraph and an additional experiment in order to address and discuss this alternative approach for evaluating the performance of BayesCCE (See the last paragraph under the subsection titled "BayesCCE impute: cell counts imputation" in the Results section). Particularly, per the reviewer's suggestion, we added a new figure which demonstrates the recovery of cell-type specific methylation profiles by BayesCCE (Supplementary Figure S8).

R1: This is already an improvement, yet from the new Supplementary Figure S8 it is very hard to judge what is going on. Can the authors perhaps answer what the purpose of smoothing is in a 5x5 matrix? Furthermore, it is quite surprising that Monocytes seemed to correlate badly with Granulocytes, an unexpected result given that they belong to the same lineage. An analogous matrix for the original reference data (it will have 1s only on the diagonal) would be useful to see how well BayesCCE results capture correlation between different cell types.

---

AU: We included two supplementary tables to fully describe the performance of each method in recovering the composition of each cell type by each one of the methods we benchmarked (Supplementary Tables T1 and T2).

R1: The tables now provide much more information to make judgments about the claimed performance of BayesCCE.

---

AU: We note that in BayeCCE, as well as in all other dimensionality reduction algorithms such as NNMF and MeDeCom, there is a distinction between the number of dimension that the algorithm emits (k), and the number of dimension that it explores. Particularly, if the original rank of the matrix is r, then all these methods provide a k-dimensional output by exploring the entire input space of dimension r > k. In other words, our use of d > k' ReFACTor PCs is still a valid and fair comparison to the other methods since these PCs are only used in the exploration stage, and we eventually output k dimensions using BayesCCE. The number of dimensions in the output is what counts, and not the number of dimensions explored by the algorithm. By allowing each method to output data with the same dimension, as we do, we allow a fair comparison in our evaluation of the different methods.

We agree that considering only k components for the reference-free methods may restrict them from capturing variation of interest. However, the focus of this paper is the scenario wherein we are interested in cell count estimates for a predefined set of cell types. Therefore, outputting a larger number of components than the number of preselected cell types would pose a serious interpretability issue. Particularly, it is not clear how this approach can be useful in real world settings, as our problem definition assumes that appropriate reference data is unavailable and selection or aggregation of components would require some sort of reference data. Moreover, we would like to note that estimating the dimension of a variation of interest in data (e.g., number of cell types) is generally an open problem. We are aware of the methods that were previously suggested for selecting the number of cell types k, however, these methods implicitly assume that the entire signal in the data consists of cell type composition variation, an assumption which clearly does not hold in reality, given the many other factors that are known to substantially contribute to variation in methylation data (e.g., age, gender, smoking, genetics, batch effects etc.).

R1: All the comments in the authors' response are valid, and the issue can be resolved if the scope of the paper is clearly and unambiguously limited to a scenario where only predefined cell types are considered. This should then be explicitly stated in all relevant sections of the manuscript, together with the fact that both reference-free methods were designed with a different primary application scenario in mind, which is supported by the fact that both methods suggested procedures for the selection of parameter k.

-----

AU: We agree with the reviewer that investigating the potential advantages of combining existing unsupervised approaches with reference methylomes is of interest. However, it is in fact the case that reference methylomes are hardly obtainable. This is probably best reflected by the fact that even though hundreds of whole-blood methylation studies have been conducted in the last few years, the go-to blood reference is still a fairly small one (n=6), which is not expected to represent every population well. This has been highlighted in a recent example with a newborn population (Yousefi et al., Environmental and molecular mutagenesis 2015). For that reason, our focus in this study is estimation of cell-type proportions without the need for methylation reference, a task which so far has not been shown to be robustly possible in real data by any method.

Moreover, we would like to note that while incorporating reference methylomes may in principle provide the same amount of information to the problem as incorporating cell counts, the latter approach is expected to be more useful in practice. Cell counts for a small fraction of the samples in a study are expected to be relatively easily obtainable, and leveraging such data is expected to allow a reasonable recovery of the cell-type-specific methylomes of the specific population in the study (matrix M). On the other hand, since obtaining reference methylomes is harder in practice (it requires cell sorting, as well as measuring the methylation levels in each cell type, which is prohibitively expensive for large samples), for most studies it would be much more reasonable to consider previously collected methylomes, which will not necessarily allow to recover important population-specific variability of the methylomes.

Finally, as implied by the reviewer, reference methylomes can be leveraged in different ways (i.e. not just the approach taken by the popular reference-based method by Houseman et al.). Yet, given such information makes the inference problem to be reference-based, as the solution depends upon the reference methylomes, rather than reference-free or semi-supervised.

Due to the above reasons, we feel that investigating the possibility of incorporating reference methylomes is out of the scope of this paper.

R1: It is fair enough that the authors decided to leave scenarios involving reference data out of scope of their paper and opt not to test them despite of possible benefits (had BayesCCE outperformed such semi-supervised extensions of NNMF/MeDeCom authors could have used it as an additional argument for their method's superiority). However, it is then unclear what the practical advantages of "BayesCCE imp" and "BayesCCE imp ext" would be compared to a simple two-stage procedure: (1) estimate cell type methylomes based on the samples with available proportions; (2) using the obtained methylomes estimate proportions in the rest of the samples via the standard reference-based method. The authors should perform such an experiment and compare the results to BayesCCE.

-----

AU: Given an optimal solution, we do not understand what would it mean to get "a more optimal solution". We kindly ask the reviewer to clarify their comment.

R1: The comment was related to one of the possible interpretations and practical implications of the proof. One such implication is that the standard NNMF procedure could be directly used to show that the solution M' (reference methylomes) and R' (their proportions, better counting-based), used as the ground truth in this study, does not belong to the set of NNMF optimal and therefore equivalent solutions. This would be required to state that this solution is in some sense qualitatively different from those returned by NNMF and MeDeCom, as it is for instance done on page 4 of the manuscript (third paragraph).

---

Given the non-identifiability nature of this problem, the approximation error of (6) is not very informative with respect to the quality of the cell count estimates. Particularly, our proof suggests that an NNMF solution with a better approximation error does not necessarily mean better cell composition estimates. Furthermore, a typical optimal solution of BayesCCE (and of MeDeCom), which takes a form of a penalized NNMF, is expected to provide worse approximation error compared with an optimal solution of a standard NNMF objective. Yet, as we show in the paper through an extensive set of experiments, BayesCCE outperform the alternatives in terms of both correlation and absolute error. This can be explained in part by the fact that the approximation error in (6) may require using sources of variation other than cell type composition in order to be minimized, and therefore a standard NNMF solution would pick such signals.

R1: Total approximation error for all cases would have been useful in order to judge where all the considered algorithms stand with respect to the issues raised in the context of non-identifiability which the authors have given so much attention. Yet it is obvious that due to the fact that each method was applied to a different set of rows of O (CpGs) the approximation errors will not be exactly comparable. To overcome this one could report RMSE of the sub-problem with fixed R solved using respective recovered proportions on the complete O. Authors should add a supplementary figure/table with this error reported for all scenarios, as well as the RMSE of (6) for the case when M contains complete reference methylomes from Reinius et al.

------

AU: Indeed, we have previously used the GALA II data set (Rahmani et al., Nature Methods 2016), in which cell counts are available for a subset of the individuals in the data. However, these cell counts were measured in a few months difference from the methylation levels, resulting in a lower accuracy of the cell count estimates by all methods. It has recently been suggested that due to this issue, the GALA II data is not ideal for benchmarking of methods for estimating cell composition (Zheng et al., Nature methods 2017, Rahmani et al., Nature methods 2017), and we therefore decided to omit this data set from our evaluation of BayesCCE, as we feel that the evidence is compelling without adding this dataset. We note that we did evaluate the performance of BayesCCE and the other reference-free methods using the GALA II data set as well, and we found BayesCCE to overall outperform the alternative methods. Specifically, considering the measured cell counts as the ground truth (k=3; only counts of granulocytes, lymphocytes and monocytes were available), we found the best performing method to be BayesCCE imp (MAC 0.46, MAE 0.015). The best performance among the alternative reference-free methods was MAC 0.381 and MAE 0.261. We further found BayesCCE imp to perform best when considering the reference-based estimates as the ground truth, with MAC 0.84 and MAE 0.015 for k=3, and MAC 0.62 and MAE 0.03 for k=6, as opposed to the best performance among the alternative reference-free methods: MAC 0.58 and MAE 0.11 for k=3, and MAC 0.37 and MAE 0.14 for k=6.

R1: Fair enough. Would this also then require an erratum to Rahmani et al, 2016?

---

AU: We agree with the reviewer that looking at biological scenarios other than whole-blood is of interest. Unfortunately, it is hard to evaluate more complex scenarios with the currently available data. Specifically, in order to evaluate BayesCCE on data from other tissues, one would need a ground truth cell-counts for the studied tissue or alternatively a reference data that has been shown to provide a good surrogate for the ground truth. To the best of our knowledge, such data is not available except for blood tissue. We therefore opt to focus on evaluating the performance of BayesCCE using multiple large whole-blood data sets - as the reviewer pointed out, the current reference data for whole-blood tissue is now acknowledged to do a decent job in estimating cell counts, and it therefore provides an opportunity to conduct a reliable benchmarking.

We would like to note that beyond its potential utility for complex biological scenarios for which reference is unavailable, BayesCCE may also provide an opportunity to improve cell count estimates in whole-blood studies in scenarios where the currently available reference data is not appropriate. Notably, in a recent work we have shown that ReFACTor outperforms the reference-based method in correction for cell composition in multiple whole-blood data sets (Rahmani et al., Nature Methods 2017). Differences in performance between ReFACTor (upon which BayesCCE relies for obtaining a starting point which captures the cell composition variation in the data) and the reference-based method are expected to be especially large in studies where the available reference data do not represent the individuals in the study well. We argue that this is likely to be typically the case, as the current go-to whole blood reference consists of only six individuals, which represent a very specific and narrow population in terms of methylome altering factors, such as genetics, age and gender. That said, large data sets with cell counts are required in order to fully investigate and demonstrate these claims.

In order to clarify these issues, we added a new paragraph to the paper (see the second to last paragraph under the Discussion section).

R1: Although the authors' desire to avoid a tedious task is understandable, it is not true that no other tissue has enough data to facilitate a similar analysis. Neuronal cell fractions presented in the MeDeCom paper is one example of a system where reference data is available. Moreover, approximate proportion of different cell types required by BayesCCE are also relatively well known for brain tissue as well as many other human tissues. One additional non-standard application scenario might have helped to attract the interest of broader readership from adjacent areas of epigenetic research, however this is fully in the hands of the authors.

More minor mistakes:

--legend Figure S8, the second to last line: "BayesCCE imp etx"

Reviewer 2:

The authors have done a very comprehensive job of responding to reviewer comments.

One minor point - the legend to Figure 5 could be improved.

**Authors’ response**

Dear Reviewers and Editors,

We thank the reviewers for their comments. We have revised the manuscript to address the reviewers’ questions and suggestions, and we believe that the manuscript has improved. We include a file where the changes in the text are highlighted in a blue font. Below we provide a point by point response to the reviewers’ comments (our responses are in regular fonts).

Reviewer #1:

-----

AU: We thank the reviewer for raising and emphasizing this important issue. We added an entirely new subsection including a set of new experiments to address and discuss the issue of potential biases introduced into BayesCCE by the prior information (see "Robustness of BayesCCE to biases introduced by the cell composition prior" under the Results section). Through a series of extensive experiments, we found BayesCCE to handle such biases remarkably well. Specifically, we included the following additional analyses:

- We verified that cell counts estimated by BayesCCE capture differences between populations (e.g., cases versus controls) even when using a single general prior to describe all the individuals in the data (Figure 5). Our new experiments demonstrate that BayesCCE leverages the prior information in a way that directs the optimization towards estimation of cell counts, while not imposing too strong constraints that would lead to missing natural cell composition variability between different populations (such as variability between cases and controls).

- We evaluated the performance of BayesCCE under different levels of noise added to the prior information (Supplementary Figure S9). As expected, we found very noisy priors to impact the performance of BayesCCE, however, even in the presence of a non-informative prior, BayesCCE overall outperformed existing reference-free methods.

- We updated our implementation to allow incorporation of more than one prior into BayesCCE. Yet, we show that incorporating two priors (e.g., a separate prior for cases and for controls) provides no improvement compared with using a single general prior (Supplementary Table T3). This further confirms our conclusion that BayesCCE handles the bias introduced by using a single prior remarkably well.

*We further added an appropriate discussion about the above issues under the Discussion section.*

R1: The experiments look convincing, good job, although it seems that proportions returned by BayesCCE can sometimes contain false-positive associations (see e.g. NK cells by basic BayesCCE in Hannum data, Monocytes by BayesCCE imp in Hannon II etc ) while also having false-negatives (Monocytes by BayesCCE imp ext in Hannum, NK cells by BayesCCE im in Hannon I etc ).

*Indeed, Figure 5 demonstrates several disagreements between the different methods. Unfortunately, since a ground truth in the form of cell counts was not available for the analysis, it is unclear how to determine which one of the methods provides a better representation of the true underlying differences in cell proportions. Particularly, the cell proportion estimates given by the reference-based approach may also demonstrate false positive or false negative changes in cell composition, which may lead to inconsistencies with the results of BayesCCE. For instance, the significant changes reported by BayesCCE for NK cells in the Hannum et al. data that were suggested by the reviewer as false positives may reflect true changes in NK proportions. In fact, NK cells abundance is known to change in aging as part of a process known as NK cell immunosenescence (e.g., Solana et al. 1999, Solana et al. 2000), therefore this result may potentially represent a false negative result for the reference-based method, rather than a false positive for BayesCCE. Another example is the results reported for the differences in monocytes between rheumatoid arthritis cases and controls (the Liu et al. data set). While the reference-based estimates revealed no difference between cases and control, the BayesCCE estimates did indicate such a difference. Since monocyte levels are known to increase in rheumatoid arthritis patients compared with healthy individuals (e.g., Kawanaka et al. 2002, Wijngaarden et al. 2003, Iwahashi et al. 2004), this result may very likely reflect again a false negative of the reference-based estimates rather than a false-positive of BayesCCE.*

*That said, there are some inconsistencies between the different BayesCCE approaches (BayesCCE, BayesCCE imp and BayesCCE imp ext), which suggest that some of the results may indeed be false positives or false negatives. Yet, we believe that the overall high agreement between the estimates of the several BayesCCE approaches and the reference-based approach indicates that the BayesCCE framework allows the detection of differences in cell composition between different populations in spite of using a single general prior as an input. Clearly, it is left of interest to quantify the extent to which each method can detect such differences accurately, however, a robust and reliable investigation of this question would only be possible given several large data sets with corresponding cell counts, which are currently unavailable.*

*In order to clarify this matter, we edited the subsection “Robustness of BayesCCE to biases introduced by the cell composition prior” (Results section); it now reads as follows:*

*“...notably, in some of the data sets we found BayesCCE to demonstrate some differences between cases and controls which were not captured by the reference-based estimates (Figure 5). For example,... These differences in cell populations were detected by BayesCCE but not by the reference-based method, thus suggesting that BayesCCE could uncover signal which was undetected by the reference-based method (Figure 5). That said, some other cell composition differences that were reported by BayesCCE but not by the reference-based method or vice versa may be the result of inaccuracies introduced by BayesCCE. Quantifying more accurately and reliably to what extent each method can detect cell composition differences would require several large data sets with known cell counts.”*

------

AU: Indeed, cell types which are not characterized by stable DNA methylation signatures that are globally separable from those of other cell types in the input data are not expected to be estimated well. This problem is clearly not unique to BayesCCE but general to any reference-free or reference-based method. For example, the reference-based method looks for distinct cell-type specific methylomes which can be used for a decomposition, as considering non-distinct methylomes (or, equivalently, cell types with no distinct signatures) will result in a non-identifiable problem. Yet, it is widely recognized that the analysis of methylation data requires a correction for cell type composition (Jaffe & Irrizari, 2012), and thus these methods are widely used despite their inherent limitation presented by the reviewer. Bounded by this clear inherent limitation of all methods for estimating cell type composition, we believe that the second argument made by the reviewer regarding the

strength of signals is of more interest.

< omitted part of the response to fit into the character limit>

*We added the following discussion to the paper (under the Discussion section):*

*"Since BayesCCE relies on the ReFACTor components, it is limited by their quality, and particularly, if the variability of some cell type is not captured by ReFACTor, BayesCCE will not be able to estimate that cell type well. Such a result is possible in scenarios where the variation of a particular cell type is substantially weaker than other sources of variation in the data (which are unrelated to cell type composition); we note, however, that this potential limitation is not exclusive for ReFACTor or BayesCCE but rather a general limitation of all existing reference-free methods."*

R1: Unfortunately, the main message of my initial comment did not get through, perhaps due to an insufficiently clear formulation. The point is that the classical cell types, usually defined via various cell surface markers, do not necessarily correspond to cell types which one would identify given sufficient knowledge about the epigenomes of all cells present in a certain tissue. Moreover, proportions and methylomes of the latter cell types will most likely appear as linear combinations of the former and vice versa. In that respect the existing reference-based methods are by design limited to recover only the proportions of surface marker-defined cell types. Formulated more directly, reference-based methods with references such as Reinius et al. (as well as analogous cell counting approaches) predict nothing but proportions of cells baring a certain surface marker. Reference-free methods have, at least, some chances to detect the true "epigenetic" cell types. A strict

benchmark of the kind the authors have performed is, therefore, only feasible iff they can show that these two cell type definitions are at least roughly equivalent. Otherwise, the lack of correspondence between the results of reference-based and reference-free methods as central argument for BayesCCE is rather weak. In any case, these issues have to be extensively covered in the Discussion.

*We thank the reviewer for clarifying this point. We agree that cell types can be defined either by using the classical definition via cell surface markers or by using other definitions such as genomic patterns (e.g., DNA methylation or gene expression), and the decision of which definition to use should be in general context-specific. It would clearly be of interest to consider methylation-based definition of cell types in the context of BayesCCE, however, the community has not settled yet on any specific definition for cell types based on methylation data. Importantly, such a definition would require obtaining appropriate single cell methylation data, which is currently scarcely available, and deriving the cell type definitions from such data is a non-trivial problem which is out of the scope of the current paper.*

*We added a new paragraph under the Discussion section in order to address this issue:*

*“The parameters of the prior required by BayesCCE can be estimated by utilizing previous studies that collected cell counts from the tissue of interest. In our evaluation of the method we used whole-blood methylation data, and we considered the classical definition of leukocyte cell types, which relies on cell surface markers. Considering other definitions of cell types is of potential interest; particularly, it would be interesting to examine whether BayesCCE can capture cell-type composition following a methylation-based definition of cell types (i.e. when defining cell types according to their methylation patterns). Since BayesCCE captures cell composition variation under the classical definition of cell types by using the most dominant components of variation in the data, the main cell types of a natural methylation-based definition are expected to be a linear combination of the cell types under the classical definition. Much like in the experiments we presented here, wherein given a prior about the distribution of the cell types BayesCCE directed the solution towards an appropriate linear transformation, we would expect BayesCCE to perform similarly in the case of a methylation-based definition of cell types (given appropriate prior information about the distribution of cell types). Nevertheless, obtaining such a definition and evaluating BayesCCE under that definition would require obtaining appropriate single cell methylation data, which is currently scarcely available. Moreover, deriving an actual meaningful definition of cell types given such data is a non-trivial problem. Therefore, until such definition and appropriate data are available, we are bounded to consider the classical definition of cell types.”*

-----

AU: We added a new paragraph and an additional experiment in order to address and discuss this alternative approach for evaluating the performance of BayesCCE (See the last paragraph under the subsection titled "BayesCCE impute: cell counts imputation" in the Results section). Particularly, per the reviewer's suggestion, we added a new figure which demonstrates the recovery of cell-type specific methylation profiles by BayesCCE (Supplementary Figure S8).

R1: This is already an improvement, yet from the new Supplementary Figure S8 it is very hard to judge what is going on. Can the authors perhaps answer what the purpose of smoothing is in a 5x5 matrix? Furthermore, it is quite surprising that Monocytes seemed to correlate badly with Granulocytes, an unexpected result given that they belong to the same lineage. An analogous matrix for the original reference data (it will have 1s only on the diagonal) would be useful to see how well BayesCCE results capture correlation between different cell types.

*We thank the reviewer for raising this issue. We followed the reviewer’s suggestion and we believe that the revised figure according to the reviewer’s recommendation nicely demonstrates the fact that BayesCCE captures the correlation between different cell types as reflected in the reference data.*

*Specifically, the purpose of the smoothing was to account for the fact that calculating a correlations matrix for the methylation reference effectively means to calculate correlations across the methylomes of only 6 individuals, the number of individuals in the Reinius et al. reference (and therefore the correlations are expected to be noisy). However, in light of the reviewer’s comment, we feel that smoothing prevents a clear interpretation of the figure. We therefore generated an alternative Figure S8. The new figure is now formatted exactly following the reviewer’s suggestion; for each data set we present a matrix with the correlations between the reference methylomes and the analogous matrix for BayesCCE using the estimated methylomes. As Figure S8 now shows, there is in fact a high similarly between the correlations captured in the reference methylomes and the correlations captured in the methylomes estimated by BayesCCE.*

*We would like to note that the correlation patterns demonstrated by the reference methylomes represent our best available knowledge about the differences between the methylomes of different cell types. Hence, we consider the correlation patterns as a given observation (and do not try to evaluate it based on biological knowledge such as the lineage of leukocytes).*

---

AU: We included two supplementary tables to fully describe the performance of each method in recovering the composition of each cell type by each one of the methods we benchmarked (Supplementary Tables T1 and T2).

R1: The tables now provide much more information to make judgments about the claimed performance of BayesCCE.

---

AU: We note that in BayeCCE, as well as in all other dimensionality reduction algorithms such as NNMF and MeDeCom, there is a distinction between the number of dimension that the algorithm emits (k), and the number of dimension that it explores. Particularly, if the original rank of the matrix is r, then all these methods provide a k-dimensional output by exploring the entire input space of dimension r > k. In other words, our use of d > k' ReFACTor PCs is still a valid and fair comparison to the other methods since these PCs are only used in the exploration stage, and we eventually output k dimensions using BayesCCE. The number of dimensions in the output is what counts, and not the number of dimensions explored by the algorithm. By allowing each method to output data with the same dimension, as we do, we allow a fair comparison in our evaluation of the different methods.

*We agree that considering only k components for the reference-free methods may restrict them from capturing variation of interest. However, the focus of this paper is the scenario wherein we are interested in cell count estimates for a predefined set of cell types. Therefore, outputting a larger number of components than the number of preselected cell types would pose a serious interpretability issue. Particularly, it is not clear how this approach can be useful in real world settings, as our problem definition assumes that appropriate reference data is unavailable and selection or aggregation of components would require some sort of reference data. Moreover, we would like to note that estimating the dimension of a variation of interest in data (e.g., number of cell types) is generally an open problem. We are aware of the methods that were previously suggested for selecting the number of cell types k, however, these methods implicitly assume that the entire signal in the*

*data consists of cell type composition variation, an assumption which clearly does not hold in reality, given the many other factors that are known to substantially contribute to variation in methylation data (e.g., age, gender, smoking, genetics, batch effects etc.).*

R1: All the comments in the authors' response are valid, and the issue can be resolved if the scope of the paper is clearly and unambiguously limited to a scenario where only predefined cell types are considered. This should then be explicitly stated in all relevant sections of the manuscript, together with the fact that both reference-free methods were designed with a different primary application scenario in mind, which is supported by the fact that both methods suggested procedures for the selection of parameter k.

*We have now included additional clarification about the fact that BayesCCE estimates only a predefined set of cell types, while reference-free methods can potentially allow the estimation of additional cell types. We note that all reference-based methods are also limited by this issue. We made edits in the text wherever appropriate - specifically, under the “Benchmarking existing reference-free methods for capturing cell type composition” and the “BayesCCE: A Bayesian semi-supervised approach for capturing cell type composition” subsections (Results section) and under the “Model” subsection (Methods section).*

-----

AU: We agree with the reviewer that investigating the potential advantages of combining existing unsupervised approaches with reference methylomes is of interest. However, it is in fact the case that reference methylomes are hardly obtainable. This is probably best reflected by the fact that even though hundreds of whole-blood methylation studies have been conducted in the last few years, the go-to blood reference is still a fairly small one (n=6), which is not expected to represent every population well. This has been highlighted in a recent example with a newborn population (Yousefi et al., Environmental and molecular mutagenesis 2015). For that reason, our focus in this study is estimation of cell-type proportions without the need for methylation reference, a task which so far has not been shown to be robustly possible in real data by any method.

Moreover, we would like to note that while incorporating reference methylomes may in principle provide the same amount of information to the problem as incorporating cell counts, the latter approach is expected to be more useful in practice. Cell counts for a small fraction of the samples in a study are expected to be relatively easily obtainable, and leveraging such data is expected to allow a reasonable recovery of the cell-type-specific methylomes of the specific population in the study (matrix M). On the other hand, since obtaining reference methylomes is harder in practice (it requires cell sorting, as well as measuring the methylation levels in each cell type, which is prohibitively expensive for large samples), for most studies it would be much more reasonable to consider previously collected methylomes, which will not necessarily allow to recover important population-specific variability of the methylomes.

Finally, as implied by the reviewer, reference methylomes can be leveraged in different ways (i.e. not just the approach taken by the popular reference-based method by Houseman et al.). Yet, given such information makes the inference problem to be reference-based, as the solution depends upon the reference methylomes, rather than reference-free or semi-supervised.

Due to the above reasons, we feel that investigating the possibility of incorporating reference methylomes is out of the scope of this paper.

R1: It is fair enough that the authors decided to leave scenarios involving reference data out of scope of their paper and opt not to test them despite of possible benefits (had BayesCCE outperformed such semi-supervised extensions of NNMF/MeDeCom authors could have used it as an additional argument for their method's superiority). However, it is then unclear what the practical advantages of "BayesCCE imp" and "BayesCCE imp ext" would be compared to a simple two-stage procedure: (1) estimate cell type methylomes based on the samples with available proportions; (2) using the obtained methylomes estimate proportions in the rest of the samples via the standard reference-based method. The authors should perform such an experiment and compare the results to BayesCCE.

*First, we would like to note that using reference methylomes will result (by definition) in a reference-based method (i.e. supervised; although potentially a different one than the currently widely applied reference-based method) rather than a semi-supervised method (that would require less external information than in the reference-based case), which is the focus in our paper.*

*Applying the two-steps approach suggested by the reviewer would be equivalent to running BayesCCE imp and BayesCCE imp ext with a non-informative prior (a scenario which we evaluated in the paper; see Supplementary Figure S9), with the exception that the latter further restricts the estimated cell proportions to be a linear transformation of the ReFACTor components. Evaluating the latter component of the model reduces to the question of how well ReFACTor captures the cell composition variability, a question which we previously investigated extensively in Rahmani et al. 2016 and in Rahmani et al. 2017 and we cover in the discussion of the current paper (third to last paragraph in the Discussion section). We agree that directly evaluating the performance without the restriction of using the ReFACTor components (i.e. the approach suggested by the reviewer) is of interest, however, since the reference-based estimates were used as the ground truth in our study, such approach is expected to result in a substantial bias. Specifically, since the cell proportions were computed using the Reinius et al. reference, using them for learning the reference methylomes will effectively result in the same Reinius et al. reference (up to some statistical error). Using this estimated reference for learning the proportions of the rest of the samples is therefore expected to artificially provide (by definition) the highest possible concordance with the ground truth (as the ground truth of the proportions was defined using this reference, given no known cell counts).*

*For completeness, we now explicitly mention the reviewer's suggestion as another possible approach for estimating proportions given known cell counts for a subset of the individuals, and we explain why it is not possible to reliably compare between the two approaches in the context of our study. We added the following under the “Imputing cell counts using a subset of samples with measured cell counts” subsection (Methods section):*

*“We note that given an estimate as described above, we can also solve directly the problem in (12) rather than the problem in (16). This approach may be more desired in cases where P does not effectively capture the cell composition variation in the data. In the context of our study, however, it is not possible to reliably evaluate the approach of solving directly the problem in (12), owing to the fact the the ground truth we set for evaluation is based on the same matrix M. Specifically, in this case, the cell proportions of the reference individuals are expected to recover the same matrix M that was used for computing the ground truth proportions of the non-reference individuals. As a result, the estimated proportions of the non-reference individuals will be exactly the ground truth that is used in the evaluation (up to a statistical error arising from the estimation of M), regardless of the true accuracy of the estimate with respect to the true M and regardless of the true accuracy of the cell proportion estimates”.*

-----

AU: Given an optimal solution, we do not understand what would it mean to get "a more optimal solution". We kindly ask the reviewer to clarify their comment.

R1: The comment was related to one of the possible interpretations and practical implications of the proof. One such implication is that the standard NNMF procedure could be directly used to show that the solution M' (reference methylomes) and R' (their proportions, better counting-based), used as the ground truth in this study, does not belong to the set of NNMF optimal and therefore equivalent solutions. This would be required to state that this solution is in some sense qualitatively different from those returned by NNMF and MeDeCom, as it is for instance done on page 4 of the manuscript (third paragraph).

*We agree with the reviewer that it is necessary to show that the truth (or at least the ground truth as it was defined in this study) does not belong to the set of NNMF optimal solutions. In fact, this is exactly what we present in Supplementary Figure 1, where we evaluate the performance of the different reference-free methods in capturing the ground truth. Importantly, all equivalent solutions of a particular NNMF solution are just a subset of all possible linear transformations of the NNMF solution. Therefore, since our evaluation in Supplementary Figure S1 is invariant to linear transformation (see the “Evaluation of performance“ subsection in the Methods section), any equivalent solution will achieve the same performance of the particular given NNMF solution (i.e. exactly what the reviewer suggested that requires verification). We would like to kindly point the reviewer to the second paragraph under the “Benchmarking existing reference-free methods for capturing cell type composition“ subsection in the Results section, where this experiment is explicitly described.*

---

*Given the non-identifiability nature of this problem, the approximation error of (6) is not very informative with respect to the quality of the cell count estimates. Particularly, our proof suggests that an NNMF solution with a better approximation error does not necessarily mean better cell composition estimates. Furthermore, a typical optimal solution of BayesCCE (and of MeDeCom), which takes a form of a penalized NNMF, is expected to provide worse approximation error compared with an optimal solution of a standard NNMF objective. Yet, as we show in the paper through an extensive set of experiments, BayesCCE outperform the alternatives in terms of both correlation and absolute error. This can be explained in part by the fact that the approximation error in (6) may require using sources of variation other than cell type composition in order to be minimized, and therefore a standard NNMF solution would pick such signals.*

R1: Total approximation error for all cases would have been useful in order to judge where all the considered algorithms stand with respect to the issues raised in the context of non-identifiability which the authors have given so much attention. Yet it is obvious that due to the fact that each method was applied to a different set of rows of O (CpGs) the approximation errors will not be exactly comparable. To overcome this one could report RMSE of the sub-problem with fixed R solved using respective recovered proportions on the complete O. Authors should add a supplementary figure/table with this error reported for all scenarios, as well as the RMSE of (6) for the case when M contains complete reference methylomes from Reinius et al.

There are different criteria that can be used for the evaluation of the different algorithms. The reviewer proposes one of them, which lines up with the NNMF objective function. By definition, the NNMF will obtain the best results as it optimizes this objective. However, we argue that this is not the relevant measure for the following reasons. As we show in the paper, there are two problems with a standard NNMF approach in the context of estimating cell composition from methylation:

1.	A methodological issue - we prove that NNMF is non-identifiable, which means that a solution to the NNMF problem may be a non-trivial (i.e. not just a mere permutation) linear transformation of the “true” NNMF decomposition.

2.	A conceptual issue - we show that solutions given by NNMF miss some of the cell composition variation that can be detected by other approaches (using ReFACTor; see Supplementary Figure S1). Importantly, the evaluation presented in Figure S1 is invariant to linear transformations, meaning that for any linear transformation of the NNMF solution (one of which would be the “true” NNMF decomposition) the correlation with the cell composition will be exactly the same. Since NNMF fails to capture some of the cell composition variation, this experiment clearly shows that the objective of minimizing the approximation error of O is merely a moderately-reasonable surrogate to the real desired objective of minimizing the approximation error of the cell composition R.

*While (1) suggests an explanation to the observed phenomena that NNMF-based solutions provide only approximations to linear combinations of the cell proportions rather than direct approximations of the cell proportions, (2) shows that the NNMF objective, i.e., minimizing the approximation error of O, is not the right objective to consider for capturing the cell composition. Thus, it is not the right objective to consider in the evaluation of methods for estimating cell composition. Since the underlying goal of these methods is to minimize the error of the cell composition estimates, a more natural evaluation criterion is the approximation error of the cell composition R upon which we base our evaluation in the paper.*

------

AU: Indeed, we have previously used the GALA II data set (Rahmani et al., Nature Methods 2016), in which cell counts are available for a subset of the individuals in the data. However, these cell counts were measured in a few months difference from the methylation levels, resulting in a lower accuracy of the cell count estimates by all methods. It has recently been suggested that due to this issue, the GALA II data is not ideal for benchmarking of methods for estimating cell composition (Zheng et al., Nature methods 2017, Rahmani et al., Nature methods 2017), and we therefore decided to omit this data set from our evaluation of BayesCCE, as we feel that the evidence is compelling without adding this dataset. We note that we did evaluate the performance of BayesCCE and the other reference-free methods using the GALA II data set as well, and we found BayesCCE to overall outperform the alternative methods. Specifically, considering the measured cell counts as the ground truth

(k=3; only counts of granulocytes, lymphocytes and monocytes were available), we found the best performing method to be BayesCCE imp (MAC 0.46, MAE 0.015). The best performance among the alternative reference-free methods was MAC 0.381 and MAE 0.261. We further found BayesCCE imp to perform best when considering the reference-based estimates as the ground truth, with MAC 0.84 and MAE 0.015 for k=3, and MAC 0.62 and MAE 0.03 for k=6, as opposed to the best performance among the alternative reference-free methods: MAC 0.58 and MAE 0.11 for k=3, and MAC 0.37 and MAE 0.14 for k=6.

R1: Fair enough. Would this also then require an erratum to Rahmani et al, 2016?

*Definitely not. First, we described this issue in the original paper of Rahmani et al., 2016. In Rahmani et al. 2016, we explicitly explain that the cell counts were measured from blood drawn at a later date than the blood samples used for probing methylation. While this has clearly affected the accuracy of the cell counts with respect to the cell proportions of the methylation samples, the cell counts are still a useful source for comparison of methods, as the noise introduced into the cell counts by the time gap is equally unknown to all methods - there is a essentially a reduction in performance for all methods. There is therefore no error in Rahmani et al., 2016. Yet, owing to a recent debate related to this issue (Rahmani et al. 2017 and Zheng et al. 2017), we decided to omit this data set from our evaluation of BayesCCE, as we feel that the evidence is compelling without adding this data set.*

---

AU: We agree with the reviewer that looking at biological scenarios other than whole-blood is of interest. Unfortunately, it is hard to evaluate more complex scenarios with the currently available data. Specifically, in order to evaluate BayesCCE on data from other tissues, one would need a ground truth cell-counts for the studied tissue or alternatively a reference data that has been shown to provide a good surrogate for the ground truth. To the best of our knowledge, such data is not available except for blood tissue. We therefore opt to focus on evaluating the performance of BayesCCE using multiple large whole-blood data sets - as the reviewer pointed out, the current reference data for whole-blood tissue is now acknowledged to do a decent job in estimating cell counts, and it therefore provides an opportunity to conduct a reliable benchmarking.

*We would like to note that beyond its potential utility for complex biological scenarios for which reference is unavailable, BayesCCE may also provide an opportunity to improve cell count estimates in whole-blood studies in scenarios where the currently available reference data is not appropriate. Notably, in a recent work we have shown that ReFACTor outperforms the reference-based method in correction for cell composition in multiple whole-blood data sets (Rahmani et al., Nature Methods 2017). Differences in performance between ReFACTor (upon which BayesCCE relies for obtaining a starting point which captures the cell composition variation in the data) and the reference-based method are expected to be especially large in studies where the available reference data do not represent the individuals in the study well. We argue that this is likely to be typically the case, as the current go-to whole blood reference consists of only six individuals, which represent a very*

*specific and narrow population in terms of methylome altering factors, such as genetics, age and gender. That said, large data sets with cell counts are required in order to fully investigate and demonstrate these claims.*

*In order to clarify these issues, we added a new paragraph to the paper (see the second to last paragraph under the Discussion section).*

R1: Although the authors' desire to avoid a tedious task is understandable, it is not true that no other tissue has enough data to facilitate a similar analysis. Neuronal cell fractions presented in the MeDeCom paper is one example of a system where reference data is available. Moreover, approximate proportion of different cell types required by BayesCCE are also relatively well known for brain tissue as well as many other human tissues. One additional non-standard application scenario might have helped to attract the interest of broader readership from adjacent areas of epigenetic research, however this is fully in the hands of the authors.

*The neural fractions data set that was analyzed in the MeDeCom paper represents a simple decomposition case of two cell types (neural and non-neural fractions; k=2). Since the proportions of each individual sum up to one, this is effectively a problem of one dimension, which makes it an easy decomposition problem and therefore less interesting in terms of methods comparison. In contrast, in the BayesCCE paper, we considered more complicated scenarios (k=3, k=6) which clearly show the differences in performance between the different methods.*

*As we explicitly mention in the Discussion section (third paragraph), we are aware that cell compositions for tissues other than blood were previously collected and studied. However, in the absence of known (or reasonably estimated) cell compositions and corresponding methylation data for these tissues, there is no way to evaluate BayesCCE and the other methods.*

More minor mistakes:

--legend Figure S8, the second to last line: "BayesCCE imp etx"

*We thank the reviewer for noticing this error (now corrected).*

Reviewer #2:

The authors have done a very comprehensive job of responding to reviewer comments.

One minor point - the legend to Figure 5 could be improved.

*We thank the reviewer for this comment. We edited the legend of figure 5 for improved clarity.*

**Third round of review**

**Reviewer 1:**

AU: Indeed, Figure 5 demonstrates several disagreements between the different methods. Unfortunately, since a ground truth in the form of cell counts was not available for the analysis, it is unclear how to determine which one of the methods provides a better representation of the true underlying differences in cell proportions. Particularly, the cell proportion estimates given by the reference-based approach may also demonstrate false positive or false negative changes in cell composition, which may lead to inconsistencies with the results of BayesCCE. For instance, the significant changes reported by BayesCCE for NK cells in the Hannum et al. data that were suggested by the reviewer as false positives may reflect true changes in NK proportions. In fact, NK cells abundance is known to change in aging as part of a process known as NK cell immunosenescence (e.g., Solana et al. 1999, Solana et al. 2000), therefore this result may potentially represent a false negative result for the reference-based method, rather than a false positive for BayesCCE. Another example is the results reported for the differences in monocytes between rheumatoid arthritis cases and controls (the Liu et al. data set). While the reference-based estimates revealed no difference between cases and control, the BayesCCE estimates did indicate such a difference. Since monocyte levels are known to increase in rheumatoid arthritis patients compared with healthy individuals (e.g., Kawanaka et al. 2002, Wijngaarden et al. 2003, Iwahashi et al. 2004), this result may very likely reflect again a false negative of the reference-based estimates rather than a false-positive of BayesCCE.

That said, there are some inconsistencies between the different BayesCCE approaches (BayesCCE, BayesCCE imp and BayesCCE imp ext), which suggest that some of the results may indeed be false positives or false negatives. Yet, we believe that the overall high agreement between the estimates of the several BayesCCE approaches and the reference-based approach indicates that the BayesCCE framework allows the detection of differences in cell composition between different populations in spite of using a single general prior as an input. Clearly, it is left of interest to quantify the extent to which each method can detect such differences accurately, however, a robust and reliable investigation of this question would only be possible given several large data sets with corresponding cell counts, which are currently unavailable.

In order to clarify this matter, we edited the subsection "Robustness of BayesCCE to biases introduced by the cell composition prior" (Results section); it now reads as follows:

"...notably, in some of the data sets we found BayesCCE to demonstrate some differences between cases and controls which were not captured by the reference-based estimates (Figure 5). For example,... These differences in cell populations were detected by BayesCCE but not by the reference-based method, thus suggesting that BayesCCE could uncover signal which was undetected by the reference-based method (Figure 5). That said, some other cell composition differences that were reported by BayesCCE but not by the reference-based method or vice versa may be the result of inaccuracies introduced by BayesCCE. Quantifying more accurately and reliably to what extent each method can detect cell composition differences would require several large data sets with known cell counts."

------

R1: The false negatives of the reference-based method might indeed be a valid explanation for the observed inconsistencies so this answer is accepted. Moreover, the authors might want to extrapolate a similar line of thinking to the comparison between BayesCCE and the reference-free methods, i.e. the results of the reference-based method might not provide for an ideal (or, in fact, provide for a quite poor) gold standard for this type of comparison.

---------

We thank the reviewer for clarifying this point. We agree that cell types can be defined either by using the classical definition via cell surface markers or by using other definitions such as genomic patterns (e.g., DNA methylation or gene expression), and the decision of which definition to use should be in general context-specific. It would clearly be of interest to consider methylation-based definition of cell types in the context of BayesCCE, however, the community has not settled yet on any specific definition for cell types based on methylation data. Importantly, such a definition would require obtaining appropriate single cell methylation data, which is currently scarcely available, and deriving the cell type definitions from such data is a non-trivial problem which is out of the scope of the current paper.

We added a new paragraph under the Discussion section in order to address this issue:

"The parameters of the prior required by BayesCCE can be estimated by utilizing previous studies that collected cell counts from the tissue of interest. In our evaluation of the method we used whole-blood methylation data, and we considered the classical definition of leukocyte cell types, which relies on cell surface markers. Considering other definitions of cell types is of potential interest; particularly, it would be interesting to examine whether BayesCCE can capture cell-type composition following a methylation-based definition of cell types (i.e. when defining cell types according to their methylation patterns). Since BayesCCE captures cell composition variation under the classical definition of cell types by using the most dominant components of variation in the data, the main cell types of a natural methylation-based definition are expected to be a linear combination of the cell types under the classical definition. Much like in the experiments we presented here, wherein given a prior about the distribution of the cell types BayesCCE directed the solution towards an appropriate linear transformation, we would expect BayesCCE to perform similarly in the case of a methylation-based definition of cell types (given appropriate prior information about the distribution of cell types). Nevertheless, obtaining such a definition and evaluating BayesCCE under that definition would require obtaining appropriate single cell methylation data, which is currently scarcely available. Moreover, deriving an actual meaningful definition of cell types given such data is a non-trivial problem. Therefore, until such definition and appropriate data are available, we are bounded to consider the classical definition of cell types."

R1: It is rewarding to see that the authors do agree on the potential existence of an alternative definition for the cell type-methylomes compared to the "classical" one, used by reference-based methods to obtain the proportion estimates. However, they have to be consistent and admit that if such a possibility exists chances are that fully reference-free methods recover this alternative ground truth instead of the "expected" one. This would have shed new light upon the authors' benchmarking results. Unfortunately, as the authors have rightfully stated in their response, there is no real possibility to unambiguously verify this using real data yet. Therefore, a couple of sentences merely discussing such a possibility would be fair and sufficient.

-----

AU: We thank the reviewer for raising this issue. We followed the reviewer's suggestion and we believe that the revised figure according to the reviewer's recommendation nicely demonstrates the fact that BayesCCE captures the correlation between different cell types as reflected in the reference data.

Specifically, the purpose of the smoothing was to account for the fact that calculating a correlations matrix for the methylation reference effectively means to calculate correlations across the methylomes of only 6 individuals, the number of individuals in the Reinius et al. reference (and therefore the correlations are expected to be noisy). However, in light of the reviewer's comment, we feel that smoothing prevents a clear interpretation of the figure. We therefore generated an alternative Figure S8. The new figure is now formatted exactly following the reviewer's suggestion; for each data set we present a matrix with the correlations between the reference methylomes and the analogous matrix for BayesCCE using the estimated methylomes. As Figure S8 now shows, there is in fact a high similarly between the correlations captured in the reference methylomes and the correlations captured in the methylomes estimated by BayesCCE.

We would like to note that the correlation patterns demonstrated by the reference methylomes represent our best available knowledge about the differences between the methylomes of different cell types. Hence, we consider the correlation patterns as a given observation (and do not try to evaluate it based on biological knowledge such as the lineage of leukocytes).

R1: The new figure brings more clarity, and it would be a great __addition__ (I apologize in case this was not clear enough) to the original Figure S8 with no smoothing in the latter, since now the comparison between the ground truth and BayesCCE has gone completely missing. To make it ultimately clear, Figure S8 needs panels c) and d), analogous to a) and b), but featuring "ref-based" vs "BayesCCE imp"/" BayesCCE impute ext". Is it possible that this is exactly what is displayed, but the axis labels are mixed up? Otherwise 8 different "Ref-based" vs "Ref-based" make little sense given the one and only Reinius et al data set? Also, please adapt the figure legend as there is no smoothing anymore.

---

AU: We have now included additional clarification about the fact that BayesCCE estimates only a predefined set of cell types, while reference-free methods can potentially allow the estimation of additional cell types. We note that all reference-based methods are also limited by this issue. We made edits in the text wherever appropriate - specifically, under the "Benchmarking existing reference-free methods for capturing cell type composition" and the "BayesCCE: A Bayesian semi-supervised approach for capturing cell type composition" subsections (Results section) and under the "Model" subsection (Methods section).

R1: Thanks, this one is resolved now.

-----

AU: First, we would like to note that using reference methylomes will result (by definition) in a reference-based method (i.e. supervised; although potentially a different one than the currently widely applied reference-based method) rather than a semi-supervised method (that would require less external information than in the reference-based case), which is the focus in our paper.

Applying the two-steps approach suggested by the reviewer would be equivalent to running BayesCCE imp and BayesCCE imp ext with a non-informative prior (a scenario which we evaluated in the paper; see Supplementary Figure S9), with the exception that the latter further restricts the estimated cell proportions to be a linear transformation of the ReFACTor components. Evaluating the latter component of the model reduces to the question of how well ReFACTor captures the cell composition variability, a question which we previously investigated extensively in Rahmani et al. 2016 and in Rahmani et al. 2017 and we cover in the discussion of the current paper (third to last paragraph in the Discussion section). We agree that directly evaluating the performance without the restriction of using the ReFACTor components (i.e. the approach suggested by the reviewer) is of interest, however, since the reference-based estimates were used as the ground truth in our study, such approach is expected to result in a substantial bias. Specifically, since the cell proportions were computed using the Reinius et al. reference, using them for learning the reference methylomes will effectively result in the same Reinius et al. reference (up to some statistical error). Using this estimated reference for learning the proportions of the rest of the samples is therefore expected to artificially provide (by definition) the highest possible concordance with the ground truth (as the ground truth of the proportions was defined using this reference, given no known cell counts).

For completeness, we now explicitly mention the reviewer's suggestion as another possible approach for estimating proportions given known cell counts for a subset of the individuals, and we explain why it is not possible to reliably compare between the two approaches in the context of our study. We added the following under the "Imputing cell counts using a subset of samples with measured cell counts" subsection (Methods section):

"We note that given an estimate as described above, we can also solve directly the problem in (12) rather than the problem in (16). This approach may be more desired in cases where P does not effectively capture the cell composition variation in the data. In the context of our study, however, it is not possible to reliably evaluate the approach of solving directly the problem in (12), owing to the fact the the ground truth we set for evaluation is based on the same matrix M. Specifically, in this case, the cell proportions of the reference individuals are expected to recover the same matrix M that was used for computing the ground truth proportions of the non-reference individuals. As a result, the estimated proportions of the non-reference individuals will be exactly the ground truth that is used in the evaluation (up to a statistical error arising from the estimation of M), regardless of the true accuracy of the estimate with respect to the true M and regardless of the true accuracy of the cell proportion estimates".

R1: While most of the response is valid, an important misunderstanding remains. Under available proportions for the alternative "2-step" procedure __directly measured proportions aka cell counts__ for a subset of observations were meant rather than computationally estimated ones. The authors' "recycled estimation" argument does not apply in this case. This issue can be dropped in case there is indeed no suitable data set with experimental cell counts for a subset of samples, although I though such a data set is the one used at least in some of the presented imputation scenarios.

-----

We agree with the reviewer that it is necessary to show that the truth (or at least the ground truth as it was defined in this study) does not belong to the set of NNMF optimal solutions. In fact, this is exactly what we present in Supplementary Figure 1, where we evaluate the performance of the different reference-free methods in capturing the ground truth. Importantly, all equivalent solutions of a particular NNMF solution are just a subset of all possible linear transformations of the NNMF solution. Therefore, since our evaluation in Supplementary Figure S1 is invariant to linear transformation (see the "Evaluation of performance" subsection in the Methods section), any equivalent solution will achieve the same performance of the particular given NNMF solution (i.e. exactly what the reviewer suggested that requires verification). We would like to kindly point the reviewer to the second paragraph under the "Benchmarking existing reference-free methods for capturing cell type composition" subsection in the Results section, where this experiment is explicitly described.

Assuming that this interpretation of the experiment related to Figure S1 is true, one question remains. How does BayesCCE perform with respect to the initial evaluation approach, i.e. linear model R^2 to correlation coefficient? Can Figure S1 be extended with the results of BayesCCE variants?

-----

AU: There are different criteria that can be used for the evaluation of the different algorithms. The reviewer proposes one of them, which lines up with the NNMF objective function. By definition, the NNMF will obtain the best results as it optimizes this objective. However, we argue that this is not the relevant measure for the following reasons. As we show in the paper, there are two problems with a standard NNMF approach in the context of estimating cell composition from methylation:

1. A methodological issue - we prove that NNMF is non-identifiable, which means that a solution to the NNMF problem may be a non-trivial (i.e. not just a mere permutation) linear transformation of the "true" NNMF decomposition.

2. A conceptual issue - we show that solutions given by NNMF miss some of the cell composition variation that can be detected by other approaches (using ReFACTor; see Supplementary Figure S1). Importantly, the evaluation presented in Figure S1 is invariant to linear transformations, meaning that for any linear transformation of the NNMF solution (one of which would be the "true" NNMF decomposition) the correlation with the cell composition will be exactly the same. Since NNMF fails to capture some of the cell composition variation, this experiment clearly shows that the objective of minimizing the approximation error of O is merely a moderately-reasonable surrogate to the real desired objective of minimizing the approximation error of the cell composition R.

While (1) suggests an explanation to the observed phenomena that NNMF-based solutions provide only approximations to linear combinations of the cell proportions rather than direct approximations of the cell proportions, (2) shows that the NNMF objective, i.e., minimizing the approximation error of O, is not the right objective to consider for capturing the cell composition. Thus, it is not the right objective to consider in the evaluation of methods for estimating cell composition. Since the underlying goal of these methods is to minimize the error of the cell composition estimates, a more natural evaluation criterion is the approximation error of the cell composition R upon which we base our evaluation in the paper.

R1: As the authors have a very solid theory behind their observed results, I now see even less reasons not to show the RMSE values since they would have helped the authors to make their point. My interest, as a potential reader, is stirred up ever more: are the RMSEs of both NNMF-like methods indeed lower as the theory dictates? A display item with contrasting RMSEs and performance metric on R for different methods (where RMSE increases along with a decreasing error on R) would have backed the theory with solid data and would have made things much clearer for many potential users. Furthermore, I find the above response much more suitable for the parts of the main text explaining the non-identifiability proof, as compared to what is there now. Taken together, to me it seems that the authors miss a perfect opportunity to present one of their major results in a clear and understandable way. It is pretty unfortunate, given that they have selected a journal with a very broad life-science readership.

------

R1: The rest of the issues can be considered resolved.

**Authors’ response**

Dear Reviewers and Editors,

We thank the reviewer for the comments and suggestions. We have revised the manuscript to address the reviewers’ questions and suggestions. Below we provide a point-by-point response.

---

Reviewer #1:

AU: Indeed, Figure 5 demonstrates several disagreements between the different methods. Unfortunately, since a ground truth in the form of cell counts was not available for the analysis, it is unclear how to determine which one of the methods provides a better representation of the true underlying differences in cell proportions. Particularly, the cell proportion estimates given by the reference-based approach may also demonstrate false positive or false negative changes in cell composition, which may lead to inconsistencies with the results of BayesCCE. For instance, the significant changes reported by BayesCCE for NK cells in the Hannum et al. data that were suggested by the reviewer as false positives may reflect true changes in NK proportions. In fact, NK cells abundance is known to change in aging as part of a process known as NK cell immunosenescence (e.g., Solana et al. 1999, Solana et al. 2000), therefore this result may potentially represent a false negative result

for the reference-based method, rather than a false positive for BayesCCE. Another example is the results reported for the differences in monocytes between rheumatoid arthritis cases and controls (the Liu et al. data set). While the reference-based estimates revealed no difference between cases and control, the BayesCCE estimates did indicate such a difference. Since monocyte levels are known to increase in rheumatoid arthritis patients compared with healthy individuals (e.g., Kawanaka et al. 2002, Wijngaarden et al. 2003, Iwahashi et al. 2004), this result may very likely reflect again a false negative of the reference-based estimates rather than a false-positive of BayesCCE.

That said, there are some inconsistencies between the different BayesCCE approaches (BayesCCE, BayesCCE imp and BayesCCE imp ext), which suggest that some of the results may indeed be false positives or false negatives. Yet, we believe that the overall high agreement between the estimates of the several BayesCCE approaches and the reference-based approach indicates that the BayesCCE framework allows the detection of differences in cell composition between different populations in spite of using a single general prior as an input. Clearly, it is left of interest to quantify the extent to which each method can detect such differences accurately, however, a robust and reliable investigation of this question would only be possible given several large data sets with corresponding cell counts, which are currently unavailable.

*In order to clarify this matter, we edited the subsection "Robustness of BayesCCE to biases introduced by the cell composition prior" (Results section); it now reads as follows:*

*"...notably, in some of the data sets we found BayesCCE to demonstrate some differences between cases and controls which were not captured by the reference-based estimates (Figure 5). For example,... These differences in cell populations were detected by BayesCCE but not by the reference-based method, thus suggesting that BayesCCE could uncover signal which was undetected by the reference-based method (Figure 5). That said, some other cell composition differences that were reported by BayesCCE but not by the reference-based method or vice versa may be the result of inaccuracies introduced by BayesCCE. Quantifying more accurately and reliably to what extent each method can detect cell composition differences would require several large data sets with known cell counts."*

------

R1: The false negatives of the reference-based method might indeed be a valid explanation for the observed inconsistencies so this answer is accepted. Moreover, the authors might want to extrapolate a similar line of thinking to the comparison between BayesCCE and the reference-free methods, i.e. the results of the reference-based method might not provide for an ideal (or, in fact, provide for a quite poor) gold standard for this type of comparison.

*We would like to kindly point the reviewer to the fourth paragraph in the Discussion, where we discuss and elaborate about the appropriateness of using the results of the reference-based method as the gold standard in our study.*

---------

We thank the reviewer for clarifying this point. We agree that cell types can be defined either by using the classical definition via cell surface markers or by using other definitions such as genomicpatterns (e.g., DNA methylation or gene expression), and the decision of which definition to use should be in general context-specific. It would clearly be of interest to consider methylation-based definition of cell types in the context of BayesCCE, however, the community has not settled yet on any specific definition for cell types based on methylation data. Importantly, such a definition would require obtaining appropriate single cell methylation data, which is currently scarcely available, and deriving the cell type definitions from such data is a non-trivial problem which is out of the scope of the current paper.

We added a new paragraph under the Discussion section in order to address this issue:

"The parameters of the prior required by BayesCCE can be estimated by utilizing previous studies that collected cell counts from the tissue of interest. In our evaluation of the method we used whole-blood methylation data, and we considered the classical definition of leukocyte cell types, which relies on cell surface markers. Considering other definitions of cell types is of potential interest; particularly, it would be interesting to examine whether BayesCCE can capture cell-type composition following a methylation-based definition of cell types (i.e. when defining cell types according to their methylation patterns). Since BayesCCE captures cell composition variation under the classical definition of cell types by using the most dominant components of variation in the data, the main cell types of a natural methylation-based definition are expected to be a linear combination of the cell types under the classical definition. Much like in the experiments we presented here,

wherein given a prior about the distribution of the cell types BayesCCE directed the solution towards an appropriate linear transformation, we would expect BayesCCE to perform similarly in the case of a methylation-based definition of cell types (given appropriate prior information about the distribution of cell types). Nevertheless, obtaining such a definition and evaluating BayesCCE under that definition would require obtaining appropriate single cell methylation data, which is currently scarcely available. Moreover, deriving an actual meaningful definition of cell types given such data is a non-trivial problem. Therefore, until such definition and appropriate data are available, we are bounded to consider the classical definition of cell types."

R1: It is rewarding to see that the authors do agree on the potential existence of an alternative definition for the cell type-methylomes compared to the "classical" one, used by reference-based methods to obtain the proportion estimates. However, they have to be consistent and admit that if such a possibility exists chances are that fully reference-free methods recover this alternative ground truth instead of the "expected" one. This would have shed new light upon the authors' benchmarking results. Unfortunately, as the authors have rightfully stated in their response, there is no real possibility to unambiguously verify this using real data yet. Therefore, a couple of sentences merely discussing such a possibility would be fair and sufficient.

*We note that the non-identifiability nature of the classical NNMF approach is independent of the definition of cell types. That said, as the reviewer suggested, it may be the case that existing reference-free methods will perform better under different definitions of cell types. We now emphasized this in the second paragraph in the Discussion section, where we discuss the possibility of alternative definitions of cell types:*

*“...Considering other definitions of cell types is of potential interest; particularly, it would be interesting to examine to what extent BayesCCE and the reference-free methods can capture cell-type composition following a methylation-based definition of cell types (i.e. when defining cell types according to their methylation patterns)...”*

-----

AU: We thank the reviewer for raising this issue. We followed the reviewer's suggestion and we believe that the revised figure according to the reviewer's recommendation nicely demonstrates the fact that BayesCCE captures the correlation between different cell types as reflected in the reference data.

Specifically, the purpose of the smoothing was to account for the fact that calculating a correlations matrix for the methylation reference effectively means to calculate correlations across the methylomes of only 6 individuals, the number of individuals in the Reinius et al. reference (and therefore the correlations are expected to be noisy). However, in light of the reviewer's comment, we feel that smoothing prevents a clear interpretation of the figure. We therefore generated an alternative Figure S8. The new figure is now formatted exactly following the reviewer's suggestion; for each data set we present a matrix with the correlations between the reference methylomes and the analogous matrix for BayesCCE using the estimated methylomes. As Figure S8 now shows, there is in fact a high similarly between the correlations captured in the reference methylomes and the correlations captured in the methylomes estimated by BayesCCE.

We would like to note that the correlation patterns demonstrated by the reference methylomes represent our best available knowledge about the differences between the methylomes of different cell types. Hence, we consider the correlation patterns as a given observation (and do not try to evaluate it based on biological knowledge such as the lineage of leukocytes).

R1: The new figure brings more clarity, and it would be a great __addition__ (I apologize in case this was not clear enough) to the original Figure S8 with no smoothing in the latter, since now the comparison between the ground truth and BayesCCE has gone completely missing. To make it ultimately clear, Figure S8 needs panels c) and d), analogous to a) and b), but featuring "ref-based" vs "BayesCCE imp"/" BayesCCE impute ext". Is it possible that this is exactly what is displayed, but the axis labels are mixed up? Otherwise 8 different "Ref-based" vs "Ref-based" make little sense given the one and only Reinius et al data set? Also, please adapt the figure legend as there is no smoothing anymore.

*We generated a new Figure S8 which includes boths evaluations and an updated legend. Note that multiple ‘Ref-based’ vs. ‘Ref-based’ evaluations are shown since in each experiment (i.e. for each data set and for each of the two approaches, BayesCCE impute and BayesCCE impute ext) a different set of features may be selected, therefore potentially leading to some (minor) differences in the estimated methylomes as given by the reference data.*

---

AU: We have now included additional clarification about the fact that BayesCCE estimates only a predefined set of cell types, while reference-free methods can potentially allow the estimation of additional cell types. We note that all reference-based methods are also limited by this issue. We made edits in the text wherever appropriate - specifically, under the "Benchmarking existing reference-free methods for capturing cell type composition" and the "BayesCCE: A Bayesian semi-supervised approach for capturing cell type composition" subsections (Results section) and under the "Model" subsection (Methods section).

R1: Thanks, this one is resolved now.

-----

AU: First, we would like to note that using reference methylomes will result (by definition) in a reference-based method (i.e. supervised; although potentially a different one than the currently widely applied reference-based method) rather than a semi-supervised method (that would require less external information than in the reference-based case), which is the focus in our paper.

Applying the two-steps approach suggested by the reviewer would be equivalent to running BayesCCE imp and BayesCCE imp ext with a non-informative prior (a scenario which we evaluated in the paper; see Supplementary Figure S9), with the exception that the latter further restricts the estimated cell proportions to be a linear transformation of the ReFACTor components. Evaluating the latter component of the model reduces to the question of how well ReFACTor captures the cell composition variability, a question which we previously investigated extensively in Rahmani et al. 2016 and in Rahmani et al. 2017 and we cover in the discussion of the current paper (third to last paragraph in the Discussion section). We agree that directly evaluating the performance without the restriction of using the ReFACTor components (i.e. the approach suggested by the reviewer) is of interest, however, since the reference-based estimates were used as the ground truth in our study, such approach is expected to result in a substantial bias. Specifically, since the cell proportions were computed using the Reinius et al. reference, using them for learning the reference methylomes will effectively result in the same Reinius et al. reference (up to some statistical error). Using this estimated reference for learning the proportions of the rest of the samples is therefore expected to artificially provide (by definition) the highest possible concordance with the ground truth (as the ground truth of the proportions was defined using this reference, given no known cell counts).

For completeness, we now explicitly mention the reviewer's suggestion as another possible approach for estimating proportions given known cell counts for a subset of the individuals, and we explain why it is not possible to reliably compare between the two approaches in the context of our study. We added the following under the "Imputing cell counts using a subset of samples with measured cell counts" subsection (Methods section):

"We note that given an estimate as described above, we can also solve directly the problem in (12) rather than the problem in (16). This approach may be more desired in cases where P does not effectively capture the cell composition variation in the data. In the context of our study, however, it is not possible to reliably evaluate the approach of solving directly the problem in (12), owing to the fact the the ground truth we set for evaluation is based on the same matrix M. Specifically, in this case, the cell proportions of the reference individuals are expected to recover the same matrix M that was used for computing the ground truth proportions of the non-reference individuals. As a result, the estimated proportions of the non-reference individuals will be exactly the ground truth that is used in the evaluation (up to a statistical error arising from the estimation of M), regardless of the true accuracy of the estimate with respect to the true M and regardless of the

true accuracy of the cell proportion estimates".

R1: While most of the response is valid, an important misunderstanding remains. Under available proportions for the alternative "2-step" procedure __directly measured proportions aka cell counts__ for a subset of observations were meant rather than computationally estimated ones. The authors' "recycled estimation" argument does not apply in this case. This issue can be dropped in case there is indeed no suitable data set with experimental cell counts for a subset of samples, although I though such a data set is the one used at least in some of the presented imputation scenarios.

-----

*We agree with the reviewer that it is necessary to show that the truth (or at least the ground truth as it was defined in this study) does not belong to the set of NNMF optimal solutions. In fact, this is exactly what we present in Supplementary Figure 1, where we evaluate the performance of the different reference-free methods in capturing the ground truth. Importantly, all equivalent solutions of a particular NNMF solution are just a subset of all possible linear transformations of the NNMF solution. Therefore, since our evaluation in Supplementary Figure S1 is invariant to linear transformation (see the "Evaluation of performance" subsection in the Methods section), any equivalent solution will achieve the same performance of the particular given NNMF solution (i.e. exactly what the reviewer suggested that requires verification). We would like to kindly point the reviewer to the second paragraph under the "Benchmarking existing reference-free methods for capturing cell*

*type composition" subsection in the Results section, where this experiment is explicitly described.*

*Assuming that this interpretation of the experiment related to Figure S1 is true, one question remains. How does BayesCCE perform with respect to the initial evaluation approach, i.e. linear model R^2 to correlation coefficient? Can Figure S1 be extended with the results of BayesCCE variants?*

*As explained and elaborated throughout the paper, BayesCCE essentially looks for a linear transformation of the ReFACTor components. Therefore, linear models using the BayesCCE components will provide the same result as linear models using the ReFACTor components (i.e. the same R^2 values), and therefore there is no benefit in adding BayesCCE to Figure S1. The key merit of BayesCCE over the existing reference-free methods is its ability to provide one component per cell type rather than a linear transformation of several components per cell type.*

-----

AU: There are different criteria that can be used for the evaluation of the different algorithms. The reviewer proposes one of them, which lines up with the NNMF objective function. By definition, the NNMF will obtain the best results as it optimizes this objective. However, we argue that this is not the relevant measure for the following reasons. As we show in the paper, there are two problems with a standard NNMF approach in the context of estimating cell composition from methylation:

1. A methodological issue - we prove that NNMF is non-identifiable, which means that a solution to the NNMF problem may be a non-trivial (i.e. not just a mere permutation) linear transformation of the "true" NNMF decomposition.

2. A conceptual issue - we show that solutions given by NNMF miss some of the cell composition variation that can be detected by other approaches (using ReFACTor; see Supplementary Figure S1). Importantly, the evaluation presented in Figure S1 is invariant to linear transformations, meaning that for any linear transformation of the NNMF solution (one of which would be the "true" NNMF decomposition) the correlation with the cell composition will be exactly the same. Since NNMF fails to capture some of the cell composition variation, this experiment clearly shows that the objective of minimizing the approximation error of O is merely a moderately-reasonable surrogate to the real desired objective of minimizing the approximation error of the cell composition R.

While (1) suggests an explanation to the observed phenomena that NNMF-based solutions provide only approximations to linear combinations of the cell proportions rather than direct approximations of the cell proportions, (2) shows that the NNMF objective, i.e., minimizing the approximation error of O, is not the right objective to consider for capturing the cell composition. Thus, it is not the right objective to consider in the evaluation of methods for estimating cell composition. Since the underlying goal of these methods is to minimize the error of the cell composition estimates, a more natural evaluation criterion is the approximation error of the cell composition R upon which we base our evaluation in the paper.

R1: As the authors have a very solid theory behind their observed results, I now see even less reasons not to show the RMSE values since they would have helped the authors to make their point. My interest, as a potential reader, is stirred up ever more: are the RMSEs of both NNMF-like methods indeed lower as the theory dictates? A display item with contrasting RMSEs and performance metric on R for different methods (where RMSE increases along with a decreasing error on R) would have backed the theory with solid data and would have made things much clearer for many potential users. Furthermore, I find the above response much more suitable for the parts of the main text explaining the non-identifiability proof, as compared to what is there now. Taken together, to me it seems that the authors miss a perfect opportunity to present one of their major results in a clear and understandable way. It is pretty unfortunate, given that they have selected a journal with a very broad life-science readership.

*We thank the reviewer for this suggestion, however, it is not entirely clear what would be considered as a reasonable comparison when it comes to RMSE, as each method applies a different feature selection (and selects a different number of features). Given that, in addition to our mathematical and empirical results showing the improvement of BayesCCE upon alternative approaches in capturing R (which was set as the goal in this study), we see very little justification for exploring other, less informative quality measurements such as the RMSE.*
